# A recurrent network model of planning explains hippocampal replay and human behavior

**DOI:** 10.1038/s41593-024-01675-7

**Published:** 2024-06-07

**Authors:** Kristopher T. Jensen, Guillaume Hennequin, Marcelo G. Mattar

**Affiliations:** 1 Computational and Biological Learning Lab, Department of Engineering, University of Cambridge, Cambridge, UK; 2 Sainsbury Wellcome Centre, University College London, London, UK; 3 Department of Cognitive Science, University of California, San Diego, CA USA; 4 Department of Psychology, New York University, New York, NY USA

**Keywords:** Network models, Cognitive control, Learning algorithms, Hippocampus

## Abstract

When faced with a novel situation, people often spend substantial periods of time contemplating possible futures. For such planning to be rational, the benefits to behavior must compensate for the time spent thinking. Here, we capture these features of behavior by developing a neural network model where planning itself is controlled by the prefrontal cortex. This model consists of a meta-reinforcement learning agent augmented with the ability to plan by sampling imagined action sequences from its own policy, which we call ‘rollouts’. In a spatial navigation task, the agent learns to plan when it is beneficial, which provides a normative explanation for empirical variability in human thinking times. Additionally, the patterns of policy rollouts used by the artificial agent closely resemble patterns of rodent hippocampal replays. Our work provides a theory of how the brain could implement planning through prefrontal–hippocampal interactions, where hippocampal replays are triggered by—and adaptively affect—prefrontal dynamics.

## Abstract (web summary)

Rational planning requires time spent thinking. Here, the authors propose a model of planning where a frontal network triggers hippocampal replay, integrating the replayed trajectory in its recurrent dynamics to improve decisions.

## Main

Humans and many other animals can adapt rapidly to new information and changing environments. Such adaptation often involves spending extended and variable periods of time contemplating possible futures before taking an action^1,2^. For example, as we prepare to go to work, temporary roadworks might require us to adapt and mentally review the available routes. Because thinking does not involve the acquisition of new information or interactions with the environment, its ubiquity for human decision-making is perhaps surprising. However, thinking allows us to perform more computations with limited information, which can improve performance on downstream tasks^3^. Because physically interacting with the environment can incur unnecessary risk or consume time and other resources, the benefits of planning often make up for the time spent on the planning process itself.

Despite a wealth of cognitive science research on the algorithmic underpinnings of planning^1,4–6^, little is known about the underlying neural mechanisms. This question has been difficult to address because of a scarcity of intracortical recordings during planning and contextual adaptation. However, recent work includes large-scale neural recordings during increasingly complex behaviors from the hippocampus and prefrontal cortex (PFC), regions known to be important for memory, decision-making and adaptation^7–13^. These studies have demonstrated the importance of the PFC for generalizing abstract task structure across contexts^10,11^. Additionally, it has been suggested that planning could be mediated by hippocampal forward replays^5,7,8,14–17^. Despite these preliminary theories, it is unclear how hippocampal replays could be integrated within the dynamics of downstream circuits to implement planning^18^.

While prevailing theories of learning from replays generally rely on dopamine-mediated synaptic plasticity^5,19,20^, it is unclear whether this process could operate sufficiently quickly to also inform online decision-making. It has recently been suggested that some forms of fast adaptation could result from recurrent meta-reinforcement learning (meta-RL)^10,21,22^, where adaptation to new tasks is directly implemented by the recurrent dynamics of the prefrontal network. The dynamics themselves are learned through gradual changes in synaptic weights, which are modified over many different environments and tasks in a slow process of RL. Such recurrent neural network (RNN)-based agents can rapidly adapt to a new task or environment with fixed weights after training by integrating their experiences into the hidden state of the RNN^10,21–24^. However, previous models are generally only capable of making instantaneous decisions and cannot improve their choices by ‘thinking’ before taking an action.

**Fig. 1:**
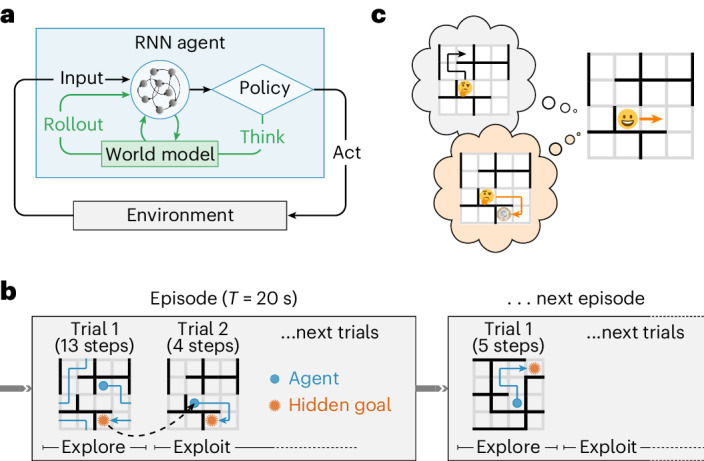
Task and model schematics. **a**, The RL agent consisted of an RNN, which received information about the environment and executed actions in response. The primary output of the agent was a policy from which the next action was sampled. This action could be to either move in the environment in a given direction (up, down, left or right) or think by using an internal world model to simulate a possible future trajectory (a rollout). The agent was trained to maximize its average reward per episode and to predict (1) the upcoming state; (2) the current goal location; and (3) the value of the current state. When the agent decided to plan, the first two predictors were used in an open-loop planning process, where the agent iteratively sampled imagined actions and predicted what the resulting state would be and whether the goal had been (virtually) reached. The output of this planning process was appended to the agent’s input on the subsequent time step (details in text). A physical action was assumed to take 400 ms and a rollout was assumed to take 120 ms (ref. ^36^). **b**, Schematic illustrating the dynamic maze task. In each episode lasting *T* = 20 s, a maze and a goal location were randomly sampled. Each time the goal was reached, the subject received a reward and was subsequently teleported to a new random location, from which it could return to the goal to receive more reward. The maze had periodic boundaries, meaning that subjects could exit one side of the maze to appear at the opposite side. **c**, Schematic illustrating how policy rollouts can improve performance by altering the momentary policy. An agent might perform a policy rollout leading to low value (top; black), which would decrease the probability of physically performing the corresponding sequence of actions. Conversely, a rollout leading to high value (bottom; orange) would increase the probability of the corresponding action sequence. Notably, these policy changes occur at the level of network dynamics rather than parameter updates (Supplementary Note 1). Source data

In this work, we propose a model that similarly combines slow synaptic learning with fast adaptation through recurrent dynamics in the prefrontal network. In contrast to previous work, however, this recurrent meta-learner can choose to momentarily forgo physical interactions with the environment and instead think (refs. ^25,26^). This process of thinking is formalized as the simulation of sequences of imagined actions, sampled from the policy of the agent itself, which we refer to as ‘rollouts’ (Fig. 1a). We introduce a flexible maze navigation task to study the relationship between the behavior of such RL agents and that of humans (Fig. 1b). RL agents trained on this task learn to use rollouts to improve their policy and selectively trigger rollouts in situations where humans also spend more time deliberating.

We draw explicit parallels between the model rollouts and hippocampal replays through reanalyses of recent hippocampal recordings from rats performing a similar maze task^7^, where the content and behavioral correlates of hippocampal replays have a striking resemblance to the policy rollouts in our computational model. Our work, thus, addresses two key questions from previous studies of hippocampal replay and planning. First, we show that a recurrent network can meta-learn when to plan instead of having to precompute a ‘plan’ to decide whether to use it^5,27^. Second, we propose a theory of replay-mediated planning, which uses fast network dynamics for real-time decision-making that could operate in parallel to slower synaptic plasticity^19^. These results provide insights into the neural underpinnings of thinking by bridging the gaps between existing research on recurrent meta-RL^10^, meta-cognition and adaptive computation^25,28–31^ and hippocampal replay for decision-making^5,15^.

## Results

### Humans think for different durations in different contexts

**Extended Data Fig. 1:**
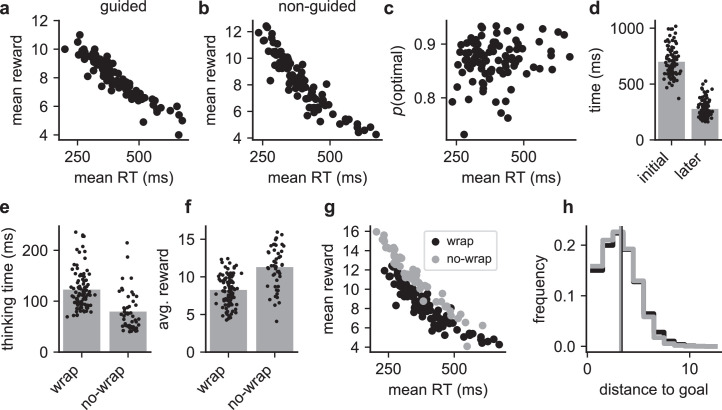
Overview of human data. **(a)** Mean reward per episode as a function of average response times during guided trials (Methods). Each data point is a single participant here and in (b)-(c). **(b)** Mean reward per episode plotted against average response times during non-guided trials. Faster participants got more reward, confirming that they were not simply making random key presses. **(c)** Fraction of actions consistent with an optimal policy plotted against mean response time during non-guided trials (*r* = 0.20; *P* = 0.024, one-sided permutation test). The positive correlation suggests that slower participants were not disengaged but instead invested more time to make higher-quality decisions. One outlier with *p*(optimal)=0.45 was excluded from this analysis. **(d)** Mean of the log-normal distribution of perception-action delays fitted to data from the guided episodes (Methods) for each participant (dots) using either the first action within each trial (left) or all other actions (right). **(e)** For comparison with the data collected with periodic maze edges, we collected data from 49 additional participants performing the same task in non-periodic mazes. These non-periodic mazes were generated such that the average shortest path length was similar to the periodic mazes considered in all other analyses (Methods). The bar plot indicates the mean across participants (black dots) of the average thinking time during non-guided exploitation trials. Thinking times were significantly higher for participants in periodic (left) than non-periodic (right) mazes (*P* < 0.001; one-sided permutation test). **(f)** Average reward per episode, which was significantly higher for participants with non-periodic than periodic boundaries (*P* < 0.001; one-sided permutation test). **(g)** Scatter plot of average reward against average response time for participants with periodic (black) or non-periodic (gray) boundaries. The results in (e)-(g) are consistent with participants having a worse ‘model-free’ policy in the unfamiliar periodic environment, which leads to both more thinking and worse performance for a given thinking time. **(h)** Histograms of pairwise distances between random start and end locations in mazes with periodic (black) or non-periodic (gray) boundaries. Vertical lines indicate the mean of each distribution. The similar distributions suggest that the effects in (e)-(g) are not due to different path lengths. Source data

**Extended Data Fig. 2:**
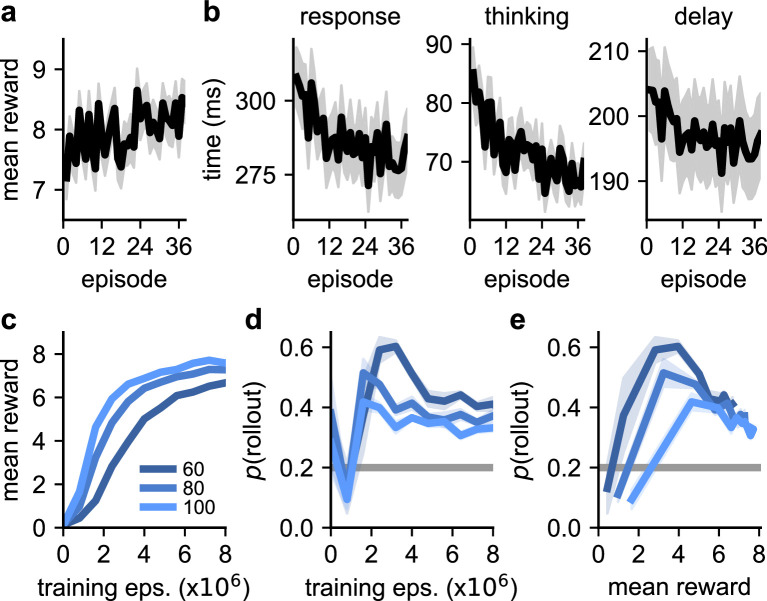
Performance and response times over learning in humans and RL agents. **(a)** Mean and standard error across human participants of the average reward for each of the 38 episodes used for all analyses (Pearson *r* = 0.075 ± 0.021; mean ± sem across participants). **(b)** Mean and standard error across participants of their median response time across episodes (left). We also plot the decomposition into thinking time (center) and sensorimotor delay (right) for each action (see Methods for details). There was a negative correlation of *r* = − 0.107 ± 0.019 (mean ± sem across participants) between episode number and response time, *r* = − 0.146 ± 0.021 for thinking time, and *r* = − 0.114 ± 0.019 for sensorimotor delay. The decrease in average median response time from the first five to the last five episodes was 6.8%, while the increase in average reward per episode was 7.6%, suggesting that a substantial part of the increase in reward could be due to faster decision making. **(c)** We trained networks of different sizes (legend; *N* ∈ {60, 80, 100}) and quantified their performance over the course of training. **(d)** Fraction of iterations where the agent performed a rollout (*p*(rollout)) at different training stages for different network sizes. The agents first learn to *suppress* the rollout frequency below chance (gray line) before increasing it again. This is consistent with rollouts only becoming useful when (i) an internal world model has been learned, and (ii) the agent has learned *how* to use rollouts to improve its policy. Rollouts become less frequent again later in training as the base policy improves, similar to how humans become faster across episodes. We hypothesize that humans start in this regime from episode 1 because they (i) construct a mental ‘world model’ immediately upon seeing the task, and (ii) already know how to integrate planning with decision making. **(e)** *p*(rollout) (panel d) as a function of the reward per episode (panel c) from the second epoch onward, showing that smaller networks also perform more rollouts after accounting for differences in training speed. Source data

To characterize the behavioral signatures of planning, we recruited 94 human participants from Prolific to perform an online maze navigation task where the walls and goal location changed periodically. The environment was a 4 × 4 grid with periodic boundaries, impassable walls and a single hidden reward (Fig. 1b and Methods; see Extended Data Fig. 1 for results with nonperiodic boundaries). The task consisted of several ‘episodes’ lasting *T* = 20 s each. At the start of each episode, the wall configuration, reward location and initial position were randomly sampled and fixed until the next episode. In the first trial, subjects explored the maze by taking discrete steps in the cardinal directions until finding the hidden reward. Subjects were then immediately moved to a new random location, initiating an exploitation phase where they had to repeatedly return to the same goal location from random start locations (Fig. 1b). Participants were paid a monetary bonus proportional to the average number of trials completed per episode (Methods and Extended Data Fig. 1) and they displayed clear signs of learning in the form of increasing reward and decreasing response times over the 40 episodes of the experiment (Extended Data Fig. 2a,b).

**Fig. 2:**
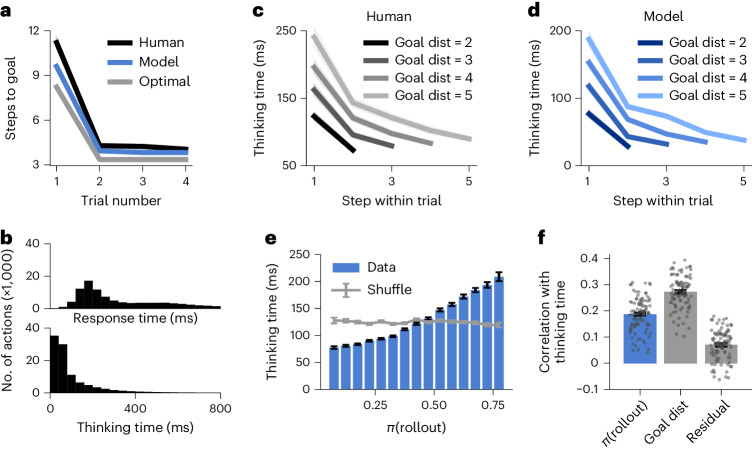
Trained RL agents perform more rollouts in situations where humans spend longer thinking. **a**, Performance (quantified as the number of actions taken to reach the goal) as a function of trial number within each episode, computed for both human participants (black) and RL agents (blue). Shading indicates the s.e.m. across human participants (*n* = 94) or RL agents (*n* = 5) and mostly falls within the interval covered by the solid lines. The gray line indicates optimal performance, computed separately for exploration (trial 1) and exploitation (trials 2–4; Methods). **b**, Distribution of human response times (top) and thinking times (bottom), spanning ranges on the order of 1 s (Methods). **c**, Human thinking time as a function of the step within trial (*x* axis) for different initial distances to the goal at the beginning of the trial (lines, legend). Shading indicates the s.e.m. across 94 participants. Participants spent more time thinking further from the goal and before the first action of each trial (Extended Data Fig. 3). **d**, Model thinking times separated by the time within trial and initial distance to goal, exhibiting a similar pattern to human participants. To compute thinking times for the model, each rollout was assumed to last 120 ms as described in the main text. Shading indicates the s.e.m. across five RL agents. The average thinking time can be less than 120 ms because the agents only perform rollouts in some instances and otherwise make a reflexive decision. This is particularly frequent near the goal and late in a trial, where humans also spend less time thinking. **e**, Binned human thinking time as a function of the probability that the agent chooses to perform a rollout, *π*(rollout). Error bars indicate the s.e.m. within each bin. The gray horizontal line indicates a shuffled control, where human thinking times were randomly permuted before the analysis. **f**, Correlation between human thinking time and the regressors (1) *π*(rollout) under the model; (2) momentary distance to goal; and (3) *π*(rollout) after conditioning on the momentary distance to goal (Residual; Methods). Bars and error bars indicate the mean and s.e.m. across human participants; gray dots indicate individual participants (*n* = 94). Source data

We first examined human performance as a function of trial number within each episode, comparing the first exploration trial to subsequent exploitation trials. Participants exhibited a rapid ‘one-shot’ transition to goal-directed navigation after the initial exploration phase (Fig. 2a, black), consistent with previous demonstrations of rapid adaptation in ‘meta-learning’ settings^10^. We next investigated the time that participants spent thinking during the exploitation phase. We estimated the ‘thinking time’ for each action as the posterior mean under a probabilistic model that decomposes the total response time for each action (Fig. 2b, top) into the sum of the thinking time (Fig. 2b, bottom) and a perception–action delay. The prior distribution over perception–action delays was estimated for each individual using a separate set of episodes, where participants were explicitly cued with the optimal path to eliminate the need for route planning (Methods and Extended Data Fig. 1). Because the first action within each trial also required participants to parse their new position in the maze, a separate prior distribution was fitted for these actions.

**Extended Data Fig. 3:**
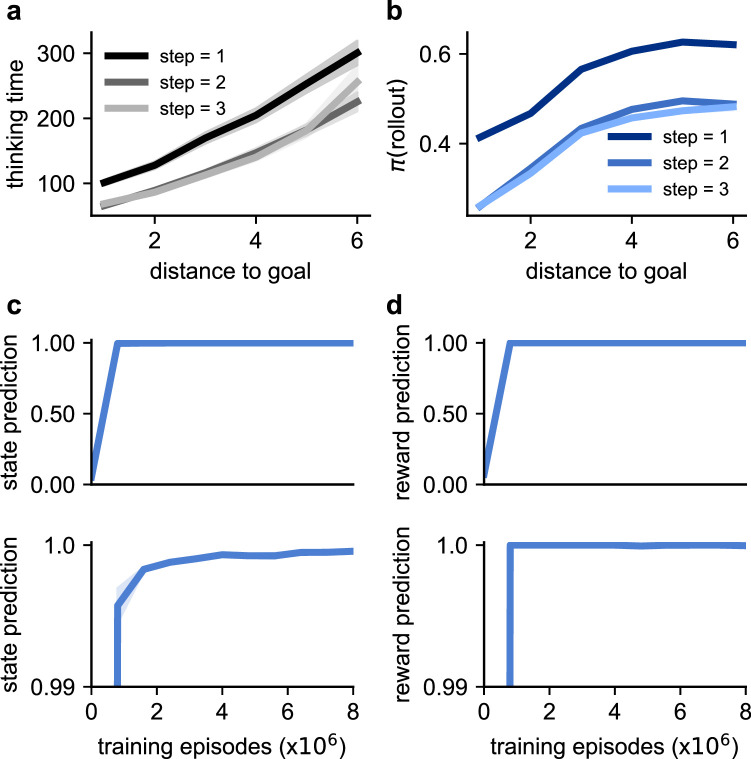
Further thinking time analyses and quantifications of internal model accuracy. **(a)** Average thinking time across human participants as a function of the momentary distance-to-goal (x-axis), conditioned on different steps within the trial (lines, legend). Subjects generally spent longer thinking before the first action of each trial, after controlling for the momentary distance-to-goal, while subsequent actions were associated with similar thinking times for a given distance-to-goal. Lines and shadings indicate mean and standard error when repeating the analysis across human participants (*n* = 94). **(b)** *π*(rollout) for the agent clamped to human trajectories as a function of the momentary distance-to-goal and step within the trial. Similar to the human participants, the agent had a higher probability of performing a rollout on the first step of each trial. Subsequent steps were associated with similar rollout probabilities after controlling for the momentary distance-to-goal. When conditioning on both momentary distance-to-goal and step within the trial, the residual correlation between *π*(rollout) and thinking time was *r* = 0.027 ± 0.004 (mean ± sem). **(c)** Accuracy of the internal transition model over the course of training. Accuracy was computed as the probability that the predicted next state was the true state reached by the agent, ignoring all teleportation steps where the transition cannot be predicted. The accuracy was averaged across all network iterations from 1,000 episodes, and the line and shading indicate mean and standard error across five RL agents. The upper panel considers the full range of [0, 1] while the lower panel considers the range [0.99, 1.0]. The transition model rapidly approaches ceiling performance, although it continues to improve slightly throughout training. **(d)** Accuracy of the internal reward model over the course of training. Accuracy was computed as the probability that the predicted reward location was the true reward location during the exploitation phase of the task (see Extended Data Fig. 7 for an analysis of the model accuracy during exploration). Lines and shadings indicate mean and standard error across five RL agents. Source data

Participants exhibited a wide distribution of thinking times during the exploitation phase (Fig. 2b, bottom). To examine task-related structure in this variability, we partitioned thinking times by within-trial action number and initial distance to the goal (Fig. 2c). Thinking times were longer when participants were further from the goal, consistent with longer routes taking longer to plan. Participants also had longer thinking times for the first action of each trial (Extended Data Fig. 3), consistent with the need to plan an entirely new route after being moved to a new location. These patterns confirm that the broad marginal distribution of thinking times (Fig. 2b) does not simply reflect a noisy decision-making process or task-irrelevant distractions. Instead, variability in thinking time is an important feature of human behavior that reflects the variable cognitive demands of action selection.

### A recurrent network model of planning

**Extended Data Fig. 4:**
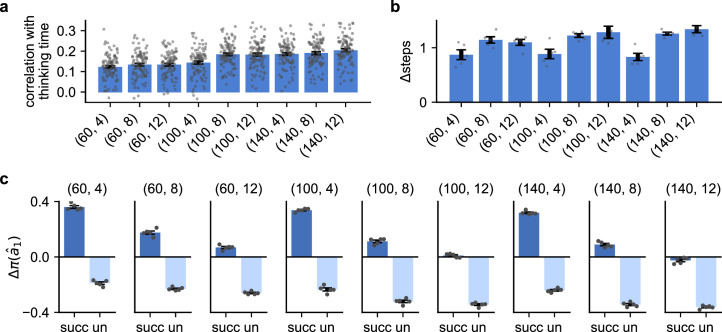
Analyses of networks with different hyperparameters. To investigate the robustness of our results to network size (*N*) and maximum planning horizon (*L*), we trained five networks with each combination of *N* ∈ {60, 100, 140} and *L* ∈ {4, 8, 12}. The results in the main text are all reported for a network with *N* = 100 and *L* = 8. **(a)** Correlation between human response times and the mean *π*(rollout) across five RL agents for each set of hyperparameters (c.f. Fig. 2f). x-ticks indicate network size and planning horizon as (*N*, *L*). Error bars indicate standard error of the mean across human participants (gray dots; *n* = 94). **(b)** Improvement in the network policy after five rollouts compared to the policy in the absence of rollouts (c.f. Fig. 3a). The policy improvement was quantified as the average number of steps needed to reach the goal on trial 2 in the absence of rollouts, minus the average number of steps needed with five rollouts enforced at the beginning of the trial. Positive values indicate that rollouts improved the policy. Bars and error bars indicate mean and standard error across five RL agents (gray dots). **(c)** For each set of hyperparameters, we computed the average change in $\pi ({\hat{a}}_{1})$ from before a rollout to after a rollout and report this change separately for successful (‘succ’) and unsuccessful (‘un’) rollouts (c.f. Fig. 3e). Positive values indicate that ${\hat{a}}_{1}$ became more likely and negative values that ${\hat{a}}_{1}$ became less likely after the rollout. Bars and error bars indicate mean and standard error across five RL agents (gray dots). Networks with longer planning horizons tend to have less positive $\Delta \pi ({\hat{a}}_{1})$ for successful rollouts and more negative $\Delta \pi ({\hat{a}}_{1})$ for unsuccessful rollouts. This is consistent with a policy gradient-like algorithm (Supplementary Note 1) with a baseline that approximates the probability of success, which increases with planning horizon. Since longer rollouts are more likely to reach the goal, we should expect them to be successful and not strongly update our policy when it occurs. Conversely, an unsuccessful rollout is less likely and should lead to a large policy change. Source data

**Extended Data Fig. 5:**
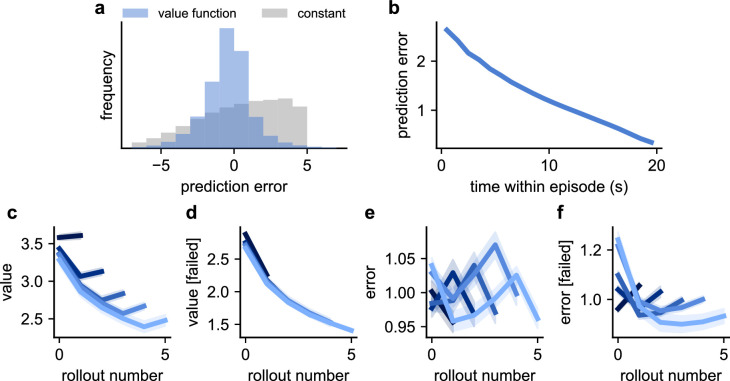
Analyses of the RL agent value function after real and imagined experience. **(a)** Blue histogram indicates the distribution of reward prediction errors across all network iterations from five RL agents, defined at each iteration as the value function of the agent minus the true reward-to-go in the episode. Gray histogram indicates a control subtracting the instantaneous reward-to-go from the mean across all agents and iterations. **(b)** Mean (line) and standard error (shading) across agents of the prediction error as a function of time within an episode. Prediction errors decrease monotonically with time since the agent is required to integrate expected reward over shorter time horizons later in the episode. The rate of decrease is fastest earlier in the episode as the agent initially does not know the goal location. **(c)** Predicted value as a function of rollout number (x-axis), plotted separately for different numbers of consecutive rollouts performed before the next physical action (colors). The darkest color corresponds to sequences of a single rollout and the lightest color to sequences of five rollouts. Lines and shadings indicate mean and standard error across five RL agents. The value function decreases with early rollouts before increasing from the very last rollout. This final increase in V could be due to successful rollouts being more likely to be followed by a physical action (Extended Data Fig. 10), making the final rollout of a sequence more likely to be successful. **(d)** As in (c), now considering only sequences of rollouts where no rollouts were successful. As expected, the predicted value did not increase for the final rollout in this setting. **(e)** Reward prediction error after a rollout as a function of rollout number. There is a decrease in prediction error after the final rollout, consistent with the increased prevalence of successful rollouts leading to a better estimate of future reward. Lines and shadings indicate mean and standard error across five RL agents. **(f)** As in (e), now considering only sequences of rollouts where no rollouts were successful. In cases where the agent performed many unsuccessful rollouts, indicating that its policy was bad, the initial value function is also likely to have been inaccurate. Source data

To model the rapid adaptation and diverse thinking times displayed by human subjects, we developed an RNN model trained in a meta-RL setting (Fig. 1a and Methods^10,21,22^; see Supplementary Note 2 for a discussion of modeling choices). The RL agent had 100 gated recurrent units (GRUs^32^; Extended Data Fig. 4) whose time-varying internal activation state *h*_k_ evolved dynamically according to

$${{{{h}}}}_{\rm{k}}={\phi }_{\theta }({{{x}}}_{\rm{k}},{{{h}}}_{\rm{k-1}})$$

$${{{y}}}_{\rm{k}}={\zeta }_{\rm \theta }({{{h}}}_{\rm{k}})$$

where *θ* denotes the model parameters, *x*_k_ denotes RNN inputs and *y*_k_ denotes its outputs. *h*_k_ was reset at the beginning of each episode. *k* indexes the evolution of the network dynamics, which can differ from the wall-clock time *t* in agents augmented with the ability to think (see below). Inputs consisted of the current agent location *s*_k_, previous action *a*_k−1_, reward *r*_k−1_, wall locations and the elapsed time *t* since the start of the episode (Methods). While the reward location was hidden and had to be discovered, the remainder of the environment was fully observed. The output consisted primarily of a policy *π*_*θ*_(*a*_k_∣*h*_k_), which was a function of the network state. At each iteration, an action *a*_k_ was sampled from *π*_*θ*_(*a*_k_∣*h*_k_). This triggered environment changes *x*_k+1_, *s*_k+1_ = *ψ*(*a*_k_, *s*_k_), which resulted in a new location *s*_k+1_ and inputs *x*_k+1_ that were fed back to the agent (Fig. 1a). In addition to the policy, the RNN output included a value function (Extended Data Fig. 5) and predictions of the agent’s next location and the current goal location (Extended Data Fig. 3).

Performance was quantified as the expected total reward according to

$$J(\theta )={{\mathbb{E}}}_{{\pi }_{\theta }}\left[\sum_{k = 1}^{K}{r}_{\rm{k}}\right]$$

where *K* denotes the number of iterations per episode, with each episode terminating when *t* exceeded *T* = 20 s as in the human data (Fig. 1b). During training, the parameters *θ* were adjusted using policy gradients to maximize the average *J*(*θ*) across environments (Methods)^10,33,34^. Because the agent lacked an intrinsic notion of wall-clock time, we considered each action to consume Δ*t* = 400 ms. This allowed 50 actions per episode, which approximately matched the human data (Supplementary Note 2).

In this canonical formulation, the RL agent takes an instantaneous action in response to its inputs, implying constant (zero) thinking time in all situations. This formulation therefore cannot explain the salient patterns of thinking times observed in human participants (Fig. 2c). At first glance, temporally extended planning might also appear unnecessary because the agent has access to all information required for decision-making, including the current state, wall configuration and reward location. However, this was also true for human participants, who spent time thinking nonetheless. We hypothesized that the RL agent could similarly benefit from the ability to trade off time for additional processing of the available information^25,26^ (Supplementary Note 2).

To test this hypothesis, we augmented the RL agent with the ability to perform temporally extended planning in the form of imagined policy rollouts. Specifically, we expanded the action space of the agent to include the option of sampling a hypothetical trajectory from its own policy (a rollout; Fig. 1a and refs. ^25,26^; see Supplementary Note 2 for a discussion of alternative planning algorithms). In other words, the agent could perform either a physical action or a mental simulation of its policy. A rollout took the form of a sequence of recurrent processing steps. At each step, the network ‘imagined’ taking an action sampled from its policy and predicted its consequences using a learned world model (Fig. 1a; see below). The world model predicted the hypothetical input to the RNN if the imagined action were actually executed from the imagined state. This predicted input was then used for the next step of recurrent processing in the rollout. The rollout process stopped after eight imagined actions or earlier if the agent imagined reaching the goal (Supplementary Note 2; see Extended Data Fig. 4 for different network sizes and planning horizons).

**Extended Data Fig. 6:**
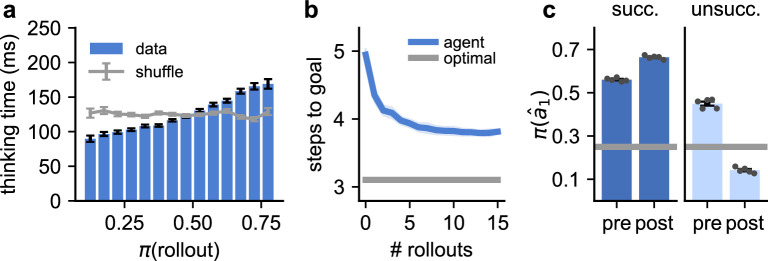
Analyses of RL agents with variable temporal opportunity costs of rollouts. In the main text, RL agents were trained with a constant temporal opportunity cost of 120 ms when performing a rollout, irrespective of the actual rollout length. In this figure, we demonstrate that our main results are not sensitive to this choice by training an additional set of agents with a ‘variable’ rollout time cost of 24 ms per imagined action. This leads to a range of rollout time costs from 24 ms to 192 ms. **(a)** Human thinking time plotted against the probability of the agent performing a rollout (*π*(rollout)) under its policy when exposed to the same mazes and action sequences as the human participants. The correlation between these two quantities was *r* = 0.099 ± 0.005 across participants. See Fig. 2e for the equivalent plot for agents trained with a constant rollout time cost. **(b)** Average number of physical actions required to reach the goal on trial 2 of an episode as a function of the number of rollouts enforced at the beginning of the episode. Blue line and shading indicate mean and standard error across five RL agents. See Fig. 3a for the equivalent plot for agents trained with a constant rollout time cost. **(c)** Probability of taking the first simulated action of the rollout, ${\hat{a}}_{1}$, before (${\pi }^{{{{\rm{pre}}}}}$ (${\hat{a}}_{1}$)) and after (*π*^post^ (${\hat{a}}_{1}$))) the rollout, evaluated separately for successful (left) and unsuccessful (right) rollouts. Error bars indicate standard error across five RL agents (dots). See Fig. 3e for the equivalent plot for agents trained with a constant rollout time cost. Source data

To capture the fact that mental simulation is faster than physical actions^35,36^, we assumed that each full rollout of up to eight imagined actions consumed only 120 ms (see Extended Data Fig. 6 for an alternative model where the temporal cost is proportional to rollout length). In other words, a single iteration of the network dynamics (*k* → *k* + 1) incremented time by 120 ms for a rollout and 400 ms for a physical action. This allowed the agent to simulate many actions in the time it would take to physically move only a short distance^14^. Importantly, because episodes had a fixed duration of 20 s, the temporal opportunity cost of rollouts resulted in less time for physical actions toward the goal.

When a rollout was performed, a flattened array of the imagined action sequence was fed back to the network as additional input for the next iteration, along with a prediction of whether the simulated action sequence reached the goal (Supplementary Note 2). These inputs affected the agent’s policy by modulating *h*_k_ through a set of learnable input weights (Fig. 1a). This is reminiscent of canonical RL algorithms that change their parameters *θ* on the basis of sampled trajectories to improve a policy. In our formulation, the policy is instead induced by the hidden state *h*_k_, which can be modulated by imagined policy rollouts to improve performance (Supplementary Note 1).

Importantly, both the generation of a rollout and the corresponding feedback relied on an internal model of the environment obtained from the agent itself. This internal model was trained alongside the policy by learning to predict the reward location and state transitions from the hidden state (*h*_k_) and action (*a*_k_) of the agent (Methods and Extended Data Fig. 3). At the beginning of each rollout, the most likely goal location according to the internal model was identified and used as an imagined goal throughout the rollout. Rollouts; therefore, did not provide any privileged information that the agent did not already possess. Instead, they allowed the agent to trade off time for additional computation—similar to thinking in humans and other animals.

Biologically, we interpret rollouts as the PFC (the RNN) interacting with the hippocampal formation (the world model) to simulate and evaluate an action sequence through replay. Following Wang et al.^10^, we use the PFC as a general term for both the PFC itself and associated areas of the striatum and thalamus (Supplementary Note 2). Importantly, while we endowed the agent with the ability to perform policy rollouts, we did not build in any knowledge of when, how or how much to use them. The agent instead learned this through training on many different environments. Therefore, while rollouts phenomenologically resembled hippocampal forward replays by design, our model allowed us to investigate (1) whether and how rollouts can drive policy improvements; (2) whether their temporal patterns explain human response times; and (3) whether biological replays might implement a similar computation.

The RL agent was trained by adjusting its parameters (*θ)* over 8 × 10^6^ episodes, sampled randomly from 2.7 × 10^8^ possible environment configurations. This large task space required the agent to generalize across tasks. Parameter adjustments followed the gradient of a cost function designed to (1) maximize expected reward; (2) learn the internal model by predicting the reward location and state transitions; and (3) maximize the policy entropy to encourage exploration (Methods)^21^. Importantly, parameters were frozen after training and the agent adapted to each new environment using only its network dynamics^10,22^.

### Human thinking times correlate with agent rollouts

Having developed a computational model of planning, we analyzed its behavior and compared it to humans. We trained five instances of the RL agent to solve the same task as human participants (Extended Data Fig. 2c). Similar to humans, the trained agents exhibited a rapid transition from exploration to exploitation upon finding the reward, reaching near-optimal performance in both phases (Fig. 2a, blue). This confirmed that these RNNs are capable of adapting to changing environments using only internal network dynamics with fixed parameters, corroborating previous work on recurrent meta-RL^10,22,37^. However, while the RL agents learned this structure through repeated exposure to the task, humans were immediately able to solve the task on the basis of written instructions (Extended Data Fig. 2a)—a potentially different type of meta-learning.

The trained networks used their capacity to perform rollouts on approximately 30% of all iterations after training (Extended Data Fig. 2d). Importantly, there was temporal variability in the probability of performing a rollout and the networks sometimes performed multiple successive rollouts between consecutive physical actions. When we queried the conditions under which the trained agents performed these rollouts, we found striking similarities with the pattern of human thinking times observed previously. In particular, the RL agent performed more rollouts earlier in a trial and further from the goal (Fig. 2d)—situations where human participants also spent more time thinking (Fig. 2c). On average, thinking times in the RL agent were approximately 50 ms lower than in humans. This difference could for example be because of (1) differences in how the periodic boundaries are represented in humans and RL agents^38^; (2) the agent having a better ‘base policy’ than humans; or (3) the hyperparameters determining the temporal cost of planning (Supplementary Note 2).

To further study the relationship between rollouts and human thinking, we simulated the RL agent in the same environments as the human participants. We did this by clamping the physical actions of the agent to those taken by the participants, while still allowing it to sample on-policy rollouts (Methods). In this setting, the agent’s probability of choosing to perform a rollout when encountering a new state, *π*(rollout), was a monotonically increasing function of human thinking time in the same situation (Fig. 2e). The Pearson correlation between these two quantities was *r* = 0.186 ± 0.007 (mean ± s.e.m. across participants), which was significantly higher than expected by chance (Fig. 2f; chance level, *r* = 0 ± 0.004). An above-chance correlation between thinking times and *π*(rollout) of *r* = 0.070 ± 0.006 persisted after conditioning on the momentary distance to goal (Fig. 2f, ‘Residual’), which was also correlated with thinking times (*r* = 0.272 ± 0.006). The similarity between planning in humans and RL agents thus extends beyond this salient feature of the task, including an increased tendency to plan on the first step of a trial (Extended Data Fig. 3).

**Extended Data Fig. 7:**
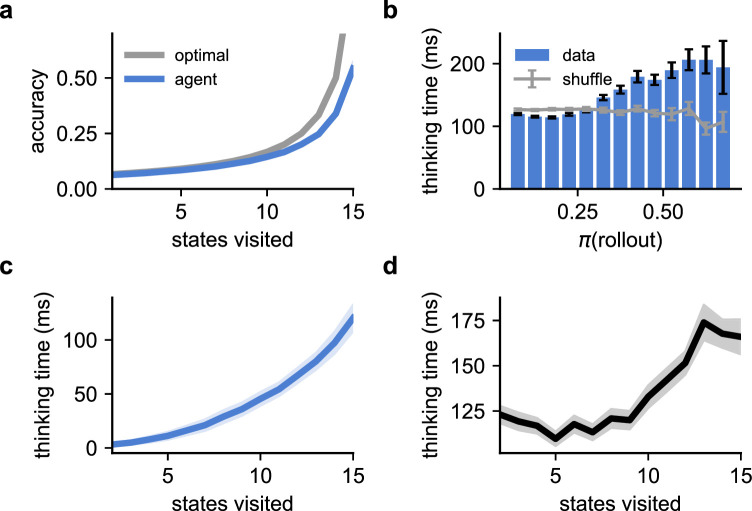
Analyses of the exploration period in humans and RL agents. **(a)** Average probability assigned to the true goal under the agent’s internal model (Methods), plotted as a function of the number of unique states visited during the exploration phase. As more states are explored, the posterior over possible goals becomes narrower and prediction accuracy increases. During a rollout, the maximum likelihood location from this posterior is used to predict the ‘success’ of the rollout, which becomes increasingly accurate as the agent explores more of the environment. This is consistent with the view of Alver and Precup^24^ that recurrent meta-reinforcement learning agents maintain a ‘belief state’ over the set of tasks they could be in, which is gradually updated based on experience. **(b)** Thinking time of human participants during exploration, plotted as a function of *π*(rollout) for RL agents clamped to the human trajectory. Bars and error bars indicate mean and standard error across all states where *π*(rollout) was in the corresponding bin. Gray line indicates a control where human thinking times have been shuffled. The Pearson correlation between *π*(rollout) and human thinking times is *r* = 0.098 ± 0.008. The very first action of the episode was not included in this or subsequent analyses of the human data. **(c)** Model thinking time as a function of the number of unique states visited during exploration, with each rollout assumed to take 120 ms (Methods). Line and shading indicate mean and standard error across five RL agents. The increase in thinking time with visited states mirrors the predictive performance from panel (a) and suggests that the agent increasingly engages in ‘model-based’ planning when the uncertainty over goal locations decreases. **(d)** Human thinking time as a function of the number of unique states visited during exploration. Line and shading indicate mean and standard error across 94 participants. The increase in thinking time with states visited suggests that humans may also transition to more model-based behavior with increasing confidence in the goal location. Source data

In addition to the similarities during the exploitation phase, a significant correlation was observed between human thinking time and *π*(rollout) during exploration (*r* = 0.098 ± 0.008). In this phase, both humans and RL agents spent more time thinking during later stages of exploration (Extended Data Fig. 7). Model rollouts during exploration correspond to planning toward an imagined goal from the posterior over goal locations, which becomes narrower as more states are explored (Extended Data Fig. 7). This finding suggests that humans may similarly engage in increasingly goal-directed behavior as the goal posterior becomes narrower over the course of exploration. In summary, a meta-RL agent, endowed with the ability to perform rollouts, learned to do so in situations similar to when humans appear to plan. This provides a putative normative explanation for the variability in human thinking times observed in the dynamic maze task.

### Rollouts improve the policy of the RL agent

In the previous section, we saw that an RL agent can learn to use policy rollouts as part of its decision-making process and that the timing and number of rollouts correlate with variability in human thinking times. We next aimed to understand why the agent chooses to perform rollouts and how they guide behavior. We considered the agent right after it first located the goal in each episode (that is, at the first iteration of trial 2; Fig. 1b) and forced it to perform a predefined number of rollouts, which we varied. We then counted the number of actions needed to return to the goal while preventing any further rollouts during this return phase (Methods).

**Fig. 3:**
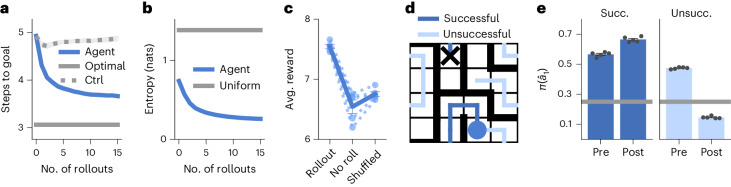
Rollouts improve the network policy. **a**, Average trial 2 performance as a function of the number of rollouts enforced at the beginning of the trial. Performance was quantified as the number of steps needed to reach the goal in the absence of further rollouts. The solid gray line (‘Optimal’) indicates optimal performance and the dashed gray line (‘Ctrl’) indicates a control simulation where the indicated number of rollouts was performed but with the feedback to the RNN from the rollout channels set to zero. The performance gap from the nonperturbed agent confirms that the performance improvement with increasing numbers of rollouts is dependent on the information contained in the rollouts and not just additional iterations of recurrent network dynamics. **b**, Policy entropy as a function of the number of rollouts enforced at the beginning of trial 2. The entropy was computed after renormalizing the policy over the four physical actions. The horizontal gray line indicates the entropy of a uniform policy. **c**, Left, original performance of the RL agent. Center, performance when renormalizing the policy over physical actions to prevent any rollouts. Right, performance after shuffling the timing of the rollouts while keeping the number of rollouts constant. Performance was quantified as the average number of rewards collected per episode. The dashed lines indicate the five individual RL agents and the solid line indicates the mean and s.e.m. across agents. Avg., average. **d**, Schematic showing an example of a successful (dark blue) and an unsuccessful (light blue) rollout from the same physical location (blue circle). The black cross indicates the goal location (not visible to the agent or human participants). **e**, Probability of taking the first simulated action of the rollout, ${\hat{a}}_{1}$, before (${\pi }^{{{{\rm{pre}}}}}({\hat{a}}_{1})$) and after (${\pi }^{{{{\rm{post}}}}}({\hat{a}}_{1})$) the rollout. This was evaluated separately for successful (left) and unsuccessful (right) rollouts. ${\pi }^{{{{\rm{pre}}}}}({\hat{a}}_{1})$ was above chance (gray line) in both cases and increased for successful rollouts, while it decreased for unsuccessful rollouts. Bars and error bars indicate the mean and s.e.m. across five agents (gray dots). The magnitude of the change in $\pi ({\hat{a}}_{1})$ for successful (Succ.) and unsuccessful (Unsucc.) rollouts depended on the planning horizon (Extended Data Fig. 4). Source data

The average number of actions needed to reach the goal decreased monotonically as the number of forced rollouts increased up to at least 15 rollouts (Fig. 3a). To confirm that this performance improvement depended on the information contained in the policy rollouts rather than being driven by additional iterations of recurrent network dynamics, we repeated the analysis with no feedback from the rollout to the RNN and found a much weaker effect (Fig. 3a, dashed gray line). The increase in performance with rollout number was also associated with a concomitant decrease in policy entropy (Fig. 3b). Thus, performing more rollouts both improved performance and reduced uncertainty (Methods). These findings confirm that the agent successfully learned to use policy rollouts to optimize its future behavior. However, the question remains of whether this policy improvement is appropriately balanced with the temporal opportunity cost of performing a rollout. In general, rollouts are beneficial in situations where the policy improvement resulting from a rollout is greater than the temporal cost of 120 ms of performing the rollout. Explicitly forbidding rollouts (Methods) impaired the performance of the agent (Fig. 3c), suggesting that it had successfully learned to trade off the cost and benefits of rollouts^14,25,26^. Randomizing the occurrence in time of the rollouts while preserving their number also led to a performance drop (Fig. 3c), confirming that the RL agent used rollouts specifically when they improved performance.

To further dissect the effect of rollouts on agent behavior, we classified each rollout, $\hat{\tau }$ (a sequence $\{{\hat{a}}_{1},{\hat{a}}_{2},\ldots \}$ of rolled-out actions), as being either ‘successful’ if it reached the goal according to the agent’s internal world model or ‘unsuccessful’ if it did not (Fig. 3d). We hypothesized that the policy improvement observed in Fig. 3a could arise from upregulating the probability of following a successful rollout and downregulating the probability of following an unsuccessful rollout. To test this hypothesis, we enforced a single rollout after the agent first found the reward and analyzed the effect of this rollout on the policy, separating the analysis by successful and unsuccessful rollouts. Importantly, we could compare the causal effect of rollout success by matching the history of the agent and performing rejection sampling from the rollout process until either a successful or an unsuccessful rollout occurred (Methods). Specifically, we asked how a rollout affected the probability of taking the first rolled-out action, ${\hat{a}}_{1}$, by comparing the value of this probability before (${\pi }^{{{{\rm{pre}}}}}({\hat{a}}_{1})$) and after (${\pi }^{{{{\rm{post}}}}}({\hat{a}}_{1})$) the rollout. ${\pi }^{{{{\rm{pre}}}}}({\hat{a}}_{1})$ was slightly higher for successful rollouts than unsuccessful rollouts, with both types of rollouts exhibiting a substantially higher-than-chance probability—a consequence of the model rollouts being ‘on-policy’ (Fig. 3e). However, while successful rollouts increased $\pi ({\hat{a}}_{1})$, unsuccessful rollouts decreased $\pi ({\hat{a}}_{1})$ (Fig. 3e). This finding demonstrates that the agent combines the spatial information of a rollout with knowledge about its consequences, based on its internal world model, to guide future behavior (Supplementary Note 1).

### Hippocampal replays resemble policy rollouts

In our computational model, we designed policy rollouts to take the form of spatial trajectories that the agent could subsequently follow and to occur only when the agent was stationary. These two properties are also important signatures of forward hippocampal replays—patterns of neural activity observed using electrophysiological recordings from rodents during spatial navigation^7–9^. We, therefore, investigated whether forward replay in biological agents could serve a similar function during decision-making to policy rollouts in the RL agent.

**Extended Data Fig. 8:**
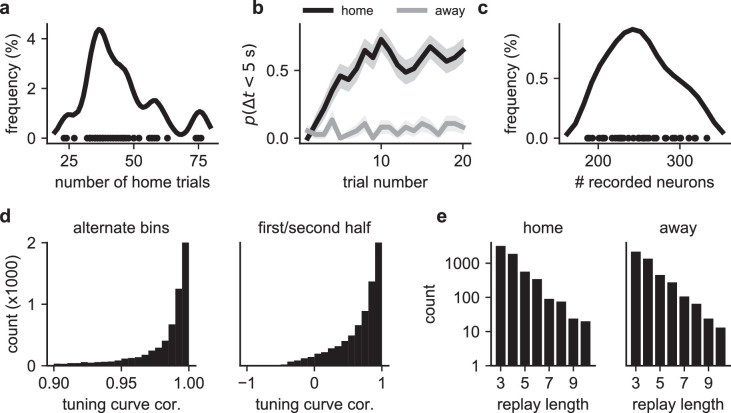
Overview of rodent data. **(a)** Kernel density estimate (*σ* = 3 trials) of the distribution of the number of ‘home’ trials in each session across all animals (an equivalent number of away trials was performed between the home trials). Dots indicate individual sessions. **(b)** Fraction of trials where the animal reached the correct goal location and started licking within 5 seconds of the trial starting, separated by home and away trials. Reaching the goal within 5 seconds was used as a success criterion by Widloski and Foster^7^ since the goal is never explicitly cued at this time (Methods). Line and shading indicate mean and standard error across sessions. The animals learn the location of the home well within a few trials and consistently return to this location on the home trials. **(c)** Distribution of the number of recorded neurons in each session. Line indicates a convolution with a Gaussian filter (15 neuron std) and dots indicate individual sessions. **(d)** Consistency of spatial tuning curves of hippocampal neurons. Consistency was quantified by constructing two tuning curves on the 5 × 5 spatial grid (Fig. 4a) for each neuron and computing the Pearson correlation between the two tuning curves. The data was split into either even/odd time bins in a session (left plot) or first/second half of the session (right plot) to compute pairs of tuning curves. **(e)** Distribution of replay lengths, measured as the number of states visited in a replay, for all replays during home (left) or away (right) trials. Note the log scale on the y-axis. Source data

**Extended Data Fig. 9:**
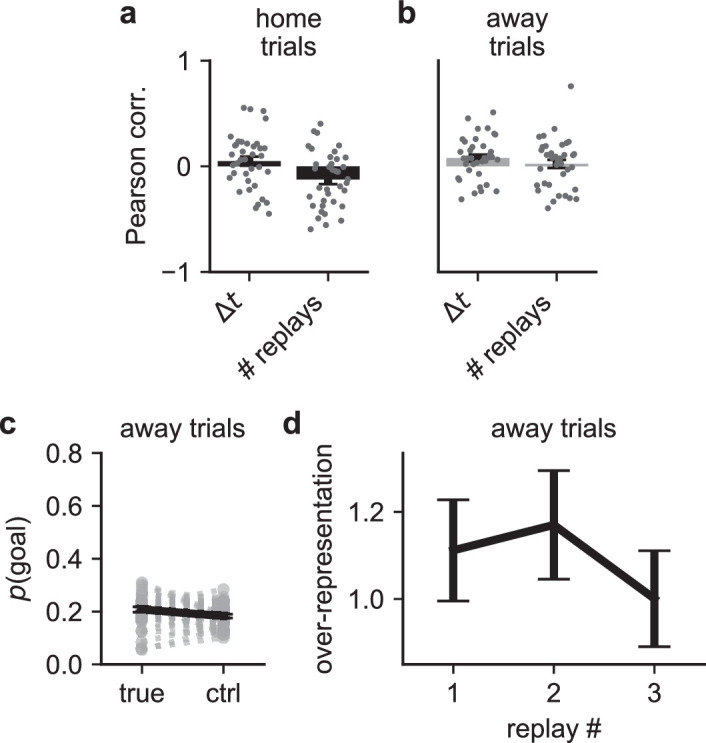
Additional analyses of the rodent dataset. For the rodent data recorded by Widloski and Foster^7^, we quantified the shortest initial distance-to-goal on each trial as well as (i) the time spent at the previous well before initiating the trial, and (ii) the number of replays detected during this period. **(a)** Pearson correlation during home trials between initial distance-to-goal and either (i) time spent at the previous well (Δ*t*, left), or (ii) the number of replays performed at the previous well (right). Bars and error bars indicate mean and standard error across sessions (gray dots; *n* = 37). The absence of a correlation between initial goal distance and time spent at the previous well differs from our analyses of human behavior in a similar maze task (Fig. 2c). However, there are two notable differences between these two paradigms that might explain the apparently discrepant results. Firstly, the rats recorded by Widloski and Foster^7^ have to physically consume the reward at the previous well before they can continue their behavior. Secondly, there is an experimenter-imposed delay between the end of reward consumption and the next reward becoming available. This is different from the human task paradigm, which was explicitly designed to encourage a trade-off between the time spent thinking and the time spent acting, without any additional ‘down time’ that could be used for planning without incurring a temporal opportunity cost. **(b)** As in (a), now for away trials. **(c)** Fraction of replays reaching either the true goal (left) or a randomly sampled alternative goal location (right) during away trials. Dashed lines indicate individual sessions (*n* = 37), and solid lines indicate mean and standard error across sessions. In contrast to the home trials (Fig. 4c), the goal is not over-represented during away trials, where the goal location is unknown. **(d)** Over-representation of replay success as a function of replay number within sequences of replays containing at least 3 distinct replay events (c.f. Fig. 4e). Bars and error bars indicate mean and standard error across replays pooled from all animals. In contrast to the home trials, there is no increase in over-representation with replay number during these away trials. Source data

To this end, we reanalyzed a recently published hippocampal dataset from rats navigating a dynamic maze similar to the task in Fig. 1b (ref. ^7^). Animals had to repeatedly return to an initially unknown ‘home’ location, akin to the goal in our task (Extended Data Fig. 8). Both this home location and the configuration of the maze changed between sessions. The rats could not be ‘teleported’ between trials as in our task; instead, they had to navigate to an unknown rewarded ‘away’ location selected at random after each home trial. These away trials served as a useful control because the animals did not know the location of the rewarded well at the beginning of the trial. Unlike the human data (Fig. 2c), we found no correlation between the initial distance to goal of the animal and time spent at the previously rewarded location (Extended Data Fig. 9). We hypothesize that this is because (1) the animals had to spend time consuming reward before they could continue and (2) a delay was imposed between reward consumption and the next reward becoming available. These periods could potentially be used for planning without incurring a substantial temporal opportunity cost, unlike the human task that explicitly enforced a trade-off between the time spent thinking and acting.

**Fig. 4:**
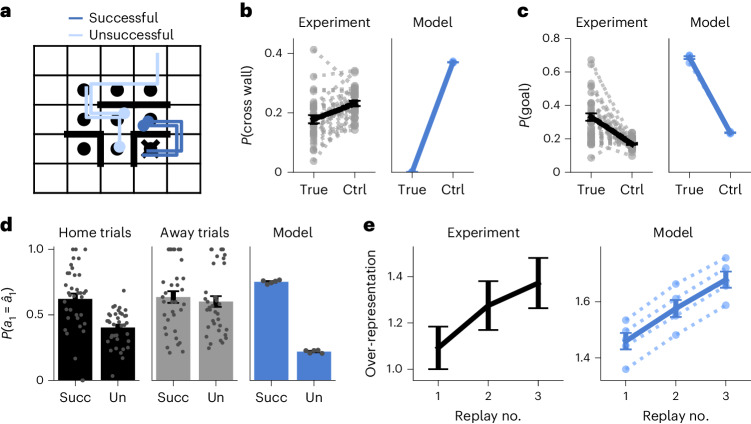
Hippocampal replays resemble model rollouts. **a**, Illustration of experimental task structure and example replays^7^. Each episode had a different wall configuration and a randomly sampled home location (cross). Between each home trial, the animal had to move to an away location, which was sampled anew on each trial (black circles). Colored lines indicate example replay trajectories originating at the blue dots. Replays were detected during the stationary periods at the away locations before returning to the home location and classified according to whether they reached the home location (dark-blue versus light-blue lines). **b**, Fraction of replay transitions that pass through a wall in the experimental (black) and model (blue) data. Control values indicate the fraction of wall crossings in resampled environments with different wall configurations. Dashed lines indicate individual biological sessions (*n* = 37) or RL agents (*n* = 5) and solid lines indicate the mean and s.e.m. across sessions or RL agents. **c**, Fraction of replays that pass through the goal location in experimental (black) and model (blue) data. Control values indicate the average fraction of replays passing through a randomly sampled nongoal location (Methods). Dashed and solid lines are as in **b**. There was no effect for the away trials, where the goal was unknown (Extended Data Fig. 9). **d**, Probability of taking the first replayed action, $P({a}_{1}={\hat{a}}_{1})$, for successful (Succ) and unsuccessful (Un) replays during home trials (left; black) and away trials (center; gray) and in the RL agent (right; blue). Bars and error bars indicate the mean and s.e.m. across sessions or RL agents (gray dots; *n* = 37 and *n* = 5, respectively). **e**, Over-representation of successful replays during trials with at least three replays in the experimental data (left) and RL agents (right). The over-representation increased with replay number, an effect not seen in the away trials (Extended Data Fig. 9). Over-representation was computed by dividing the success frequency by a reference frequency computed for randomly sampled alternative hypothetical goal locations. Bars and error bars indicate the mean and s.e.m. across replays pooled from all animals (left) or standard error across five RL agents (right; dashed lines). Source data

We, thus, focused on the spatiotemporal content of hippocampal replays following previous hypotheses that they could form a neural substrate of planning^7,8,15^. We studied replay events detected in hippocampal recordings made with tetrode drives during the maze task (*n* ∈ [187, 333] simultaneously recorded neurons per session; Extended Data Fig. 8c). To detect replays, we followed Widloski and Foster^7^ and first trained a Bayesian decoder to estimate the animal’s position on a discretized grid from the neural data during epochs when the animal was moving. We then applied this decoder during epochs when the animal was stationary at a reward location before initiating a new trial and defined replays as consecutive sequences of at least three adjacent decoded grid locations (Fig. 4a and Extended Data Fig. 8; see Methods for details).

Similar to previous work^7^, we found that the hippocampal replays avoided passing through walls to a greater extent than expected by chance (Fig. 4b; *P* < 0.001, permutation test). This finding suggests that hippocampal replays are shaped by a rapidly updating internal model of the environment, similar to how forward rollouts in the RL agent are shaped by its internal world model (Fig. 1a). Additionally, the goal location was over-represented in the hippocampal replays, consistent with the assumption of on-policy rollouts in the RL agent (Fig. 4c; *P* < 0.001, permutation test)^7^.

Inspired by our findings in the RL agent, we investigated whether a replayed action was more likely to be taken by the animal if the replay was successful than if it was unsuccessful. Here, we defined a successful replay as one that reached the goal location without passing through a wall (Fig. 4a). Consistent with the RL model, the first simulated action in biological replays agreed with the next physical action more often for successful replays than for unsuccessful replays (Fig. 4d, black; *P* < 0.001, permutation test). Such an effect was not observed in the away trials (Fig. 4d, gray; *P* = 0.129, permutation test), where the animals had no knowledge of the reward location and therefore could not know what constituted a successful replay. These findings are consistent with the hypothesis that successful replays should increase the probability of taking the replayed action, while unsuccessful replays should decrease this probability.

In the RL agent, we had direct access to the momentary policy and could quantify the causal effect of a replay on behavior (Fig. 3e). In the biological circuit, it is unknown whether the increased probability of following the first action of a successful replay is because the replay altered the policy (as in the RL agent) or because the baseline policy was already more likely to reach the goal before the replay. To circumvent this confound, we analyzed consecutive replays while the animal remained stationary. If our hypotheses hold that (1) hippocampal replays resemble on-policy rollouts of an imagined action sequence and (2) performing a replay improves the policy, then consecutive replays should become increasingly successful even in the absence of any behavior between the replays.

To test this prediction, we considered trials where the animal performed a sequence of at least three replays at the away location before moving to the home location. We then quantified the fraction of replays that were successful as a function of the replay index within the sequence, after regressing out the effect of time (Methods)^39^. We expressed this quantity as the degree to which the true goal was over-represented in the replay events by dividing the fraction of successful replays by a baseline calculated from the remaining nongoal locations, such that an over-representation of 1 implies that a replay was no more likely to be successful than expected by chance. This over-representation increased with each consecutive replay during the home trials (Fig. 4e, left) and both the second and third replays exhibited substantially higher over-representation than the first replay (*P* = 0.068 and *P* = 0.009, respectively; permutation test; Methods). Such an effect was not seen during the away trials, where the rewarded location was not known to the animal (Extended Data Fig. 9).

**Extended Data Fig. 10:**
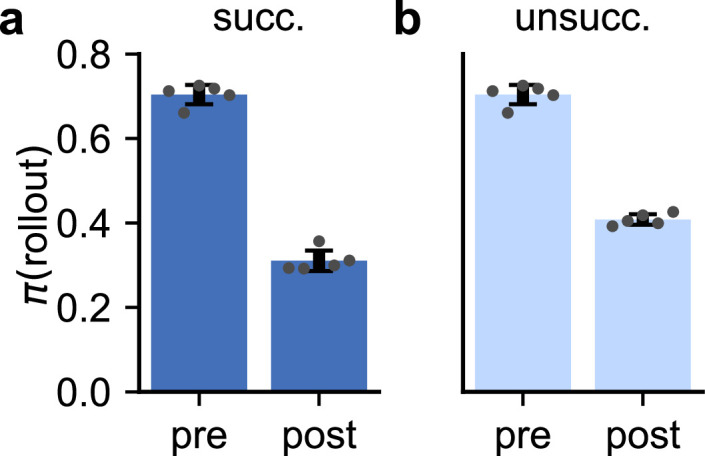
Change in *π*(rollout) after successful and unsuccessful rollouts. **(a)** *π*(rollout) before (left) and after (right) successful rollouts. Bars and error bars indicate mean and standard error across five RL agents (gray dots). The data used for this analysis was the same data used in Fig. 3E. **(b)** As in (a), now for unsuccessful rollouts. *π*^post^ (rollout) was substantially larger after unsuccessful than successful rollouts (Δ*π*^post^ (rollout)=0.10 ± 0.01; mean ± sem). Source data

These findings are consistent with a theory in which replays represent on-policy rollouts that are used to iteratively refine the agent’s policy, which in turn improves the quality of future replays—a phenomenon also observed in the RL agent (Fig. 4e, right). In the RL agent, this effect could arise in part because the agent is less likely to perform an additional rollout after a successful rollout than after an unsuccessful rollout (Extended Data Fig. 10). To eliminate this confound, we drew two samples from the policy each time the agent chose to perform a rollout and we used one sample to update the hidden state of the agent, while the second sample was used to compute the goal over-representation (Methods). Such decoupling is not feasible in the experimental data because we cannot read out the ‘policy’ of the animal. This leaves open the possibility that the increased goal over-representation with consecutive biological replays is in part because of a reduced probability of performing an additional replay after a successful replay. However, we note that (1) the rodent task was not a ‘reaction time task’ because a delay of 5–15 s was imposed between the end of reward consumption and the next reward becoming available. This makes a causal effect of replay success on the total number of replays less likely. (2) if such an effect did exist, that is also consistent with a theory where hippocampal replays guide planning.

## Discussion

We developed a theory of planning in the prefrontal–hippocampal network, implemented as an RNN model and instantiated in a spatial navigation task requiring multistep planning (Fig. 1). This model consists of a recurrent meta-RL agent augmented with the ability to plan using policy rollouts and it explains the structure observed in human behavior (Fig. 2). Our results suggest that mental rollouts could play a major role in the striking human ability to adapt rapidly to new tasks by facilitating behavioral optimization without the potential cost of executing suboptimal actions. Because mental simulation is generally faster and less risky than executing physical actions^40^, this can improve overall performance despite the temporal opportunity cost of planning (Fig. 3)^14^^,^^25^.

Our theory also suggests a role for hippocampal replays during sequential decision-making. A reanalysis of rat hippocampal recordings during a navigation task showed that patterns of hippocampal replays and their relationship to behavior resembled those of rollouts in our model (Fig. 4). These results suggest that hippocampal forward replays could be a core component of planning and that the mechanistic insights derived from our model could generalize to biological circuits. In particular, we hypothesize that forward replays should affect subsequent behavior differently depending on whether they lead to high-value or low-value states (Fig. 3)^13^, consistent with previous models where replays update state-action values to improve future behavior^5^. We suggest that forward replays could implement planning through feedback to the PFC, which drives a ‘hidden state optimization’ reminiscent of recent models of motor preparation (Supplementary Note 1)^41^. This model-based policy refinement differs from prior work that posited an arbitration between model-free and model-based policies computed separately^42,43^. Instead, we hypothesize that model-based computations iteratively update a single policy that can be used for decision-making at different stages of refinement. This is consistent with previous work proposing that model-based computations can iteratively refine values or world models learned through model-free mechanisms^5,44,45^.

### Neural mechanisms of planning and decision-making

Our model raises several hypotheses about neural dynamics in the hippocampus and PFC and how these dynamics affect behavior. One is that hippocampal replays should causally affect animal behavior, as also suggested in previous work^7,8,15^. This has been difficult to test experimentally due to the confound of how the behavioral intentions and history of an animal affect replay content^15^. Perhaps more interestingly, we predict that hippocampal replays should directly affect PFC representations, consistent with previous work showing coordinated activity between the hippocampus and PFC during sharp-wave ripples^12^. Specifically, PFC activity should change to make replayed actions more likely if the replayed trajectory is better than expected and less likely if worse than expected, reminiscent of actor–critic algorithms in the RL literature (Supplementary Note 1). These predictions can be investigated in experiments that record neural activity simultaneously from the hippocampus and PFC, where both the timing and the qualitative change in PFC representations can be related to hippocampal replays. This would be most natural in rodent experiments with electrophysiology, although human experiments using magnetoencephalography for replay detection could also investigate the effect of replays on cortical representations and behavior^35,36,46^.

While we propose a role of hippocampal replays in shaping immediate behavior through recurrent network dynamics, this is compatible with replays also having other functions over longer timescales, such as memory consolidation^47,48^ or dopamine-driven synaptic plasticity^19,20^. Additionally, we considered only the case of local forward replays and showed that they can be used to drive improved decision-making. These replays will have high ‘need’ according to the theory of Mattar and Daw^5^ because they start at the current agent location and visit likely upcoming states. They should similarly have a high ‘gain’ because rollouts lead to an increase in expected future reward (Fig. 3a). However, our choice to focus on local replays in this work does not imply that nonlocal or reverse replays could not play a similar role. Backward planning from a goal location is, for example, more efficient in environments where the branching factor is larger in the forward than the reverse direction and branching rollouts were shown to improve performance in previous RL models^26^.

### Hippocampal replay and theta sequences

This work focuses on the trade-off between thinking and acting, investigating internal computations that can improve decision-making without additional physical experience. This is the phenomenon we investigated in human behavior, where the analyses focused on stopping times. It is also an explicit feature of the RL agent, which chooses between acting and performing a rollout rather than doing both simultaneously. A putative neural correlate of such planning in the absence of behavior is hippocampal replay, given the ubiquitous finding that it occurs primarily when animals are stationary^15^ and its hypothesized role in decision-making^5,7,8,15^. While some have challenged these ideas^9,49,50^, our analyses show that a replay-like mechanism could, in principle, improve decision-making in a manner consistent with human behavior.

Another phenomenon suggested to play a role in decision-making is that of hippocampal theta sequences^17,51,52^. Theta sequences typically represent states in front of the animal^17^ and are affected by the current goal location^52^, similar to our analyses of hippocampal replays (Fig. 4). However, because theta sequences predominantly occur during active behavior, they are potentially less relevant than hippocampal replays for the trade-off between acting and thinking. Nonetheless, our RL model also suggests a potential mechanism by which short theta sequences could guide behavior by providing recurrent feedback to cortical decision-making systems about immediately upcoming states and decision points. Under this hypothesis, hippocampal replays could support longer-term planning during stationary periods while theta sequences would update these plans on the go through short-term predictions, with both types of sequences operating through recurrent feedback to cortex.

### Why do we spend time thinking?

Despite our results showing that humans and RL agents make extensive use of planning, mental simulation does not generate fundamentally new information. In theory, it should therefore be possible to make equally good ‘reflexive’ decisions given enough experience and computational power. However, previous work showed that imagination can affect human choices^53^ and that having more time to process the available information can improve decisions^54^. This raises questions about the computational mechanisms underlying this process and why decision-making often takes time instead of being instantaneous. One reason could be that our decision-making system is capacity limited and lacks the computational power to instantly generate the optimal policy^27^. This possibility is supported by our findings that smaller RNNs often perform more rollouts than larger RNNs (Extended Data Fig. 2). Another possibility is that the networks are data limited and have not received enough training to learn the optimal policy. This possibility is supported by our findings that networks of all sizes perform more rollouts early in training and gradually transition to a more reflexive policy as they experience more training data (Extended Data Fig. 2).

We hypothesize that data limitations are a major reason for the use of temporally extended planning in animals because learning the instantaneous mapping from states to actions for reflexive decisions would likely require prohibitive amounts of experience. Indeed, training our meta-reinforcement learner required millions of episodes, while humans performed well immediately after seeing a simple description and demonstration (Extended Data Fig. 2). Such rapid learning could be because of the use of generic planning algorithms as a form of ‘canonical computation’ that generalizes across tasks. When combined with a new task-specific transition function learned from relatively little experience or inferred from sensory inputs, planning would facilitate data-efficient RL by trading off processing time for a better policy^55^. This is in contrast to our current model, which had to learn from scratch both the structure of the environment and how to use rollouts to shape its behavior. Importantly, planning as a canonical computation could be generalized not only to other navigation tasks but also to other domains, such as compositional reasoning and sequence learning, where replay was recently demonstrated in humans^35,56^.

## Methods

### Software

All models were trained in Julia version 1.7 using Flux and Zygote for automatic differentiation^57^. Human behavioral experiments were written in OCaml 5.0, with the front end transpiled to JavaScript for running in the participants’ browsers. All analyses of the models and human data were performed in Julia version 1.8. All analyses of hippocampal replay data were performed in Python 3.8.

### Statistics

Unless otherwise stated, all plots are reported as the mean and s.e.m. across human participants (*n* = 94), independently trained RL agents (*n* = 5) or experimental sessions in rodents (*n* = 37).

### Environment

We generated mazes using Algorithm 1.

#### Algorithm 1

**Maze-generating algorithm** 
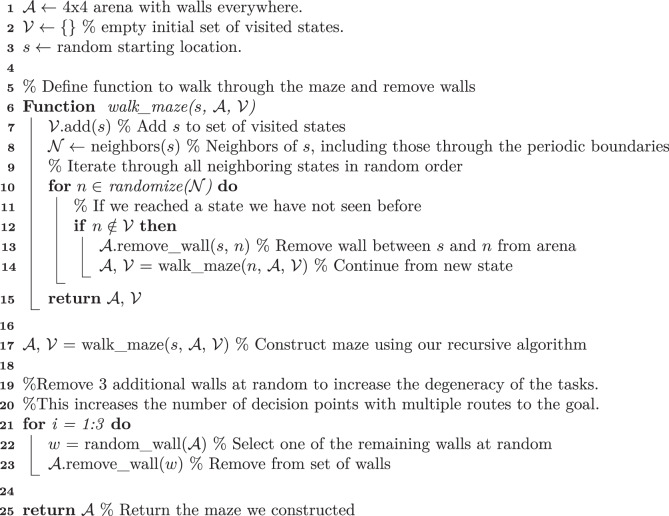


Mazes without periodic boundaries (Extended Data Fig. 1) were generated in the same way, except that states were not considered neighbors across a boundary, and four walls were removed instead of three walls in the last step of the algorithm to approximately match the distributions of shortest paths between pairs of states (Extended Data Fig. 1h).

For each environment, a goal location was sampled uniformly at random. When subjects took an action leading to the goal, they transitioned to this location before being teleported to a random location. In the computational model, this was achieved by feeding the agent an input at this location before teleporting the agent to the new location. The policy of the agent at this iteration of the network dynamics was ignored because the agent was teleported rather than taking an action.

### RL model

We trained our agent to maximize the expected reward, with the expectation taken both over environments ${{{\mathcal{E}}}}$ and the agent’s policy *π*:

$${{{\mathcal{U}}}}={{\mathbb{E}}}_{{{{\mathcal{E}}}}}\left[J(\theta )\right]$$

$$={{\mathbb{E}}}_{{{{\mathcal{E}}}}}\left[{{\mathbb{E}}}_{\pi }\left(\mathop{\sum}\limits_{k=1}^{K}{r}_{\rm{k}}\right)\right]$$

Here, ${{{\mathcal{U}}}}$ is the utility function, *k* indicates the iteration within an episode and *r*_k_ indicates the instantaneous reward at each iteration. We additionally introduced the following auxiliary losses:

$${{{{\mathcal{L}}}}}_{\rm{V}}=0.5{({V}_{\rm{k}}-{R}_{\rm{k}})}^{2}\,\,{{{\rm{value}}}}\,{{{\rm{function}}}}$$

$${{{{\mathcal{L}}}}}_{\rm{H}}={{\mathbb{E}}}_{\pi }\log \pi \,\,{{{\rm{entropy}}}}\,{{{\rm{regularization}}}}$$

$${{{{\mathcal{L}}}}}_{P}=-\sum\limits_{i}\left[{s}_{k+1}^{(i)}\log {\hat{s}}_{k+1}^{(i)}+{g}^{(i)}\log {\hat{g}}_{k}^{(i)}\right]\,\,{{{\rm{internal}}}}\,{{{\rm{world}}}}\,{{{\rm{model}}}}.$$

Here, ${\hat{g}}_{\rm{k}}$, and ${\hat{s}}_{{{\rm{k}}+1}}$ are additional network outputs containing the agent’s estimate of the current reward location and upcoming state, represented as categorical distributions. *g* and *s*_k+1_ are the corresponding ground-truth quantities, represented as one-hot vectors. ${R}_{\rm{k}}:= \mathop{\sum }\nolimits_{\rm{k}^{{\prime} } = k}^{K}{r}_{\rm{k}^{{\prime} }}$ is the empirical cumulative future reward from iteration *k* onward and *V*_k_ is the value function of the agent.

To maximize the utility and minimize the losses, we trained the RL agent on-policy using a policy gradient algorithm with a baseline^33,34^ and parameter updates of the form

$${{{{\Delta}}}} \theta \propto \sum\limits_{{ {\rm{a}}_{\rm{k}} \sim \pi}}\left[( \underbrace{\nabla_\theta \log {\pi}_k({\rm{a}_{\rm{k}})}}_{{{\rm{actor}}}} + \underbrace{\beta_v \nabla_{\theta} V_k}_{{{\rm{critic}}}} ) \delta_k - \beta_e \nabla_{\theta} \sum\limits_a \underbrace{\pi_{\rm{k,a}} \log \pi_{\rm{k,a}}}_{{{\rm{entropy}}}} + \underbrace{\beta_p {{{{\Delta}}}} \theta_{\rm{p}}}_{{{\rm{predictive}}}}\right]$$

Here, *δ*_k_ ≔ − *V*_k_ + *R*_k_ is the ‘advantage function’ and ${{\Delta }}{\theta }_{\rm{p}}={\nabla }_{\theta }{{{{\mathcal{L}}}}}_{\rm{P}}$ is the derivative of the predictive loss ${{{{\mathcal{L}}}}}_{P}$, which was used to train the ‘internal model’ of the agent. *β*_*p*_ = 0.5, *β*_*v*_ = 0.05 and *β*_*e*_ = 0.05 are hyperparameters controlling the importance of the three auxiliary losses. While we use the predictive model explicitly in the planning loop, similar auxiliary losses are also commonly used to speed up training by encouraging the learning of useful representations^58^.

Our model consisted of a GRU network with 100 hidden units^32^ (Supplementary Note 2). The policy was computed as a linear function of the hidden state followed by a softmax normalization. The value function was computed as a linear function of the hidden state. The predictions of the next state and reward location were computed with a neural network that received as input a concatenation of the current hidden state *h*_k_ and the action *a*_k_ sampled from the policy (as a one-hot representation). The output layer of this feedforward network was split into a part that encoded a distribution over the predicted next state (a vector of 16 grid locations with softmax normalization) and a part that encoded the predicted reward location in the same way. This network had a single hidden layer with 33 units and a rectified linear nonlinearity.

The model was trained using Adam^59^ on 200,000 batches, each consisting of 40 episodes, for a total of 8 × 10^6^ training episodes. These episodes were sampled independently from a total task space of (273 ± 13) × 10^6^ tasks (mean ± s.e.m.). The total task space was estimated by sampling 50,000 wall configurations and computing the fraction of the resulting 1.25 × 10^9^ pairwise comparisons that were identical, divided by 16 to account for the possible reward locations. This process was repeated ten times to estimate a mean and confidence interval. These considerations suggest that the task coverage during training was ~2.9%, which confirms that the majority of tasks seen at test time are novel (although we do not enforce this explicitly).

For all evaluations of the model, actions were sampled greedily rather than on-policy unless otherwise stated. This was done because the primary motivation for using a stochastic policy is to explore the space of policies to improve learning. Performance was better under the greedy policy at test time.

#### Planning

Our implementation of planning in the form of policy rollouts is described in Algorithm 2. This routine was invoked whenever a rollout was sampled from the policy instead of a physical action.

##### Algorithm 2

**Planning routine for the RL agent** 
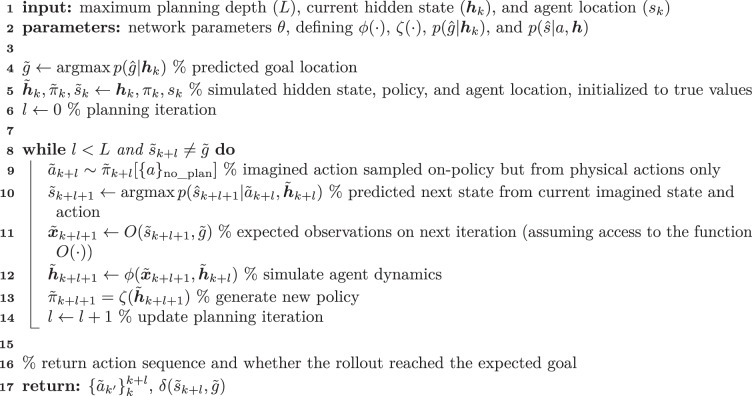


For the network update following a rollout, the input *x*_k+1_ was augmented with an additional ‘rollout input’ consisting of (1) the sequence of simulated actions, each as a one-hot vector, and (2) a binary input indicating whether the imagined sequence of states reached the imagined goal location. Additionally, the time within the session was updated by only 120 ms after a rollout in contrast to the 400-ms update after a physical action or teleportation step. For the analyses with a variable temporal opportunity cost of rollouts (Extended Data Fig. 6), we incremented time by *l* ⋅ 24 ms after a rollout, where *l* is the number of simulated actions. In Algorithm 2, we assume access to a function $O({\tilde{s}}_{\rm{k+l+1}},\tilde{g})$, which returns imagined inputs ${\tilde{{{x}}}}_{\rm{k+l+1}}$. This function is the same as that used to generate inputs from the environment, which means that we assume that the ‘predicted’ input to the RNN during a rollout takes the same form as the ‘sensory’ input from the real environment following an action.

While both an imagined ‘physical state’ ${\tilde{s}}_{\rm{k}}$ and ‘hidden state’ ${\tilde{{{h}}}}_{\rm{k}}$ are updated during the rollout, the agent continues from the original location *s*_k_ and hidden state *h*_k_ after the rollout but with an augmented input. Additionally, gradients were not propagated through the rollout process, which was considered part of the ‘environment’. This means that there was no explicit gradient signal that encouraged the policy to drive useful or informative rollouts. Instead, the rollout process simply relied on the utility of the base policy optimized for acting in the environment.

#### Performance by number of rollouts

To quantify the performance as a function of the number of planning steps in the RL agent (Fig. 3a), we simulated each agent in 1,000 different mazes until it first found the goal and was teleported to a random location. We then proceeded to enforce a particular number of rollouts before the agent was released in trial 2. During this release phase, no more rollouts were allowed; in other words, the policy was renormalized over the physical actions and the probability of performing a rollout was set to zero. Performance was then quantified as the average number of steps needed to reach the goal during this test phase. For the control without feedback, we repeated this analysis with all feedback from the rollouts set to zero, while the recurrent dynamics were allowed to proceed as usual. The optimal reference value was computed as the average optimal path length for the trial 2 starting states.

When performing more than one sequential rollout before taking an action, the policy of the agent can continue to change through two potential mechanisms. The first is that the agent can explicitly ‘remember’ the action sequences from multiple rollouts and somehow arbitrate between them. The second is to progressively update the hidden state in a way that leads to a better expected policy with each rollout because the feedback from a rollout is incorporated into the hidden state that induces the policy used to draw the next rollout. On the basis of the analysis in Supplementary Note 1, we expect the second mechanism to be dominant, although we did not explicitly test the ability of the agent to remember multiple action sequences from sequential rollouts. For these and all other RNN analyses, the agent executed the most likely action under the policy during ‘testing’ in contrast to the sampling performed during training, where such stochasticity is necessary for exploring the space of possible actions. All results were qualitatively similar if actions were sampled during the test phase, although average performance was slightly worse.

#### Performance in the absence of rollouts and with shuffled rollout times

To quantify the performance of the RL agent in the absence of rollouts, we let the agent receive inputs and produce outputs as normal. However, we set the probability of performing a rollout under the policy to zero and renormalized the policy over the physical actions before choosing an action from the policy. We compared the average performance of the agent (number of rewards collected) in this setting to the performance of the default agent in the same environments.

To compare the original performance to an agent with randomized rollout times, we counted the number of rollouts performed by the default agent in each environment. We then resampled a new set of network iterations at which to perform rollouts, matching the size of this new set to the original number of rollouts performed in the corresponding environment. Finally, we let the agent interact with the environment again, while enforcing a rollout on these network iterations and preventing rollouts at all other time steps. It is worth noting that we could not predict a priori the iterations at which the agent would find the goal, at which point rollouts were not possible. If a rollout was sampled at such an iteration, we resampled this rollout from the set of remaining network iterations.

#### Rollouts by network size

To investigate how the frequency of rollouts depended on network size (Extended Data Fig. 2), we trained networks with 60, 80 or 100 hidden units (GRUs). Five networks were trained of each size. At regular intervals during training, we tested the networks on a series of 5,000 mazes and computed (1) the average reward per episode and (2) the fraction of actions that were rollouts rather than physical actions. We then plotted the rollout fraction as a function of average reward to see how frequently an agent of a given size performed rollouts for a particular performance.

#### Effect of rollouts on agent policy

To quantify the effect of rollouts on the policy of the agent, we simulated each agent in 1,000 different mazes until it first found the goal and was teleported to a random location. We then resampled rollouts until both a successful rollout and an unsuccessful rollout had been sampled. Finally, we quantified ${\pi }^{{{{\rm{pre}}}}}({\hat{a}}_{1})$ and ${\pi }^{{{{\rm{post}}}}}({\hat{a}}_{1})$ separately for the two scenarios and plotted the results in Fig. 3e. Importantly, this means that each data point in the successful analysis had a corresponding data point in the unsuccessful analysis with the exact same maze, location and hidden state. In this way, we could query the effect of rollouts on the policy without the confound of how the policy itself affects the rollouts. For this analysis, we discarded episodes where the first 100 sampled rollouts did not result in both a successful and an unsuccessful rollout.

For Extended Data Fig. 10, we used the same episodes and instead quantified *π*(rollout) before and after the rollout, repeating the analysis for both successful and unsuccessful rollouts.

#### Overlap between hidden state updates and policy gradients (Supplementary Note 1)

Using a single rollout ($\hat{\tau }$) to approximate the expectation over trajectories of the gradient of the expected future reward for a given episode, ∇_*h*_*J*_fut_(*h*), the policy gradient update in *h* takes the form ${{\Delta }}{{h}}\propto ({R}_{\hat{\tau }}-b){\nabla }_{\rm{{h}}}\log P(\hat{\tau })$. Here, Δ*h* is the change in hidden state resulting from the rollout, ${R}_{\hat{\tau }}$ is the ‘reward’ of the simulated trajectory, *b* is a constant or state-dependent baseline and ${\nabla }_{{{h}}}\log P(\hat{\tau })$ is the gradient with respect to the hidden state of the log probability of $\hat{\tau }$ under the policy induced by *h*. This implies that the derivative of the hidden state update with respect to ${R}_{\hat{\tau }}$, ${{{{\alpha }}}}^{{{{\rm{RNN}}}}}:= \frac{\partial {{\Delta }}{{h}}}{\partial {R}_{\hat{\tau }}}$, should be proportional to ${{{{\alpha }}}}^{{{{\rm{PG}}}}}:= {\nabla }_{\rm{{h}}}\log P(\hat{\tau })$.

For these analyses, we divided $\hat{\tau }$ into its constituent actions, defining ${{{{\alpha }}}}_{\rm{k}}^{{{{\rm{PG}}}}}:= {\nabla }_{\rm{{h}}}\log p({\hat{a}}_{\rm{k}}| {\hat{a}}_{\rm{1:k-1}})$ as the derivative with respect to the hidden state of the log probability of taking the simulated action at step *k*, conditioned on the actions at all preceding steps (1 to *k* − 1) being consistent with the rollout. To compute *α*^RNN^, we also needed to take derivatives with respect to ${R}_{\hat{\tau }}$—the reward of a rollout. A naive choice here would be to simply consider ${R}_{\hat{\tau }}$ to be the input specifying whether the rollout reached the reward. However, we hypothesized that the agent would also use information about, for example, how long the simulated trajectory was in its estimate of the ‘goodness’ of a rollout (because a shorter rollout implies that the goal was found more quickly). We, therefore, determined the direction in planning input state space that was most predictive of the time to goal of the agent. We did this by using linear regression to predict the (negative) time to next reward as a function of the planning feedback *x*_*f*_ across episodes and rollouts. This defines the (normalized) direction ${\hat{\nu}}$ in planning input space that maximally increases the expected future reward. Finally, we defined ${R}_{\hat{\tau }}$ as the magnitude of the planning input in direction ${\hat{\nu}}$, ${R}_{\hat{\tau}}:= {{{x}}}_{\rm{f}}\cdot {\hat{\nu}}$. We could then compute *α*^RNN^ with this definition of ${R}_{\hat{\tau }}$ using automatic differentiation.

In Supplementary Information, Figure S1c, we computed *α*^RNN^ and ${{{{\alpha }}}}_{1}^{{{{\rm{PG}}}}}$ across 1,000 episodes and subtracted the mean across rollouts for each feature. We then performed principal component analysis on the set of ${{{{\alpha }}}}_{1}^{{{{\rm{PG}}}}}$ and projected both *α*^RNN^ and ${{{{\alpha }}}}_{1}^{{{{\rm{PG}}}}}$ into the space spanned by the top three principal components (PCs). Finally, we computed the mean value of both quantities conditioned on ${\hat{a}}_{1}$ to visualize the alignment. In Supplementary Information, Figure S1d, we considered the same ***α***^RNN^ and ${{{{\alpha }}}}_{1}^{{{{\rm{PG}}}}}$ vectors. After mean subtraction for each feature and normalization across features for each *α*, we projected these into the space spanned by the top three PCs of ${{{{\alpha }}}}_{1}^{{{{\rm{PG}}}}}$. Finally, we computed the average across rollouts of the cosine similarity between the pairs of ${{{{\alpha }}}}_{1}^{{{{\rm{PG}}}}}$ and ${{{{\alpha }}}}_{1}^{{{{\rm{RNN}}}}}$ in this latent space. We performed this analysis in a low-dimensional space because we were primarily interested in changes to *h* within the subspace that would affect $\log P(\hat{\tau })$. As a control, we repeated the analysis after altering the planning input *x*_*f*_ to falsely inform the agent that it had simulated some other action ${\hat{a}}_{1,{{{\rm{ctrl}}}}}\ne {\hat{a}}_{1}$. Finally, we also repeated this analysis using ${{{{\alpha }}}}_{2}^{{{{\rm{PG}}}}}$ to characterize how the effects of the planning input propagated through the recurrent network dynamics to modulate future action probabilities.

#### Quantification of value functions

To quantify the error of the value function in Extended Data Fig. 5, we compared the value function computed by the agent (*V*_k_) to the true reward-to-go (${R}_{\rm{k}}={\sum }_{\rm{k}^{{\prime} }\ > \ = k}{r}_{\rm{k}^{{\prime} }}$). Extended Data Fig. 5a shows the distribution of errors *V*_k_ − *R*_k_, while the ‘constant control’ shows the distribution of ${\bar{R}}_{\rm{k}}-{R}_{\rm{k}}$, where ${\bar{R}}_{\rm{k}}$ is the mean reward-to-go across all trials and iterations. These distributions were aggregated across all agents. In Extended Data Fig. 5c, we considered sequences of *n* consecutive rollouts and computed the average value function before the first rollout and after each rollout. Extended Data Fig. 5d further conditions on all the rollouts in a sequence being unsuccessful.

### Human data collection

The human behavioral experiments used in this study were certified as exempt from institutional review board review by the University of California San Diego Human Research Protection Program. We collected data from 100 human participants (50 male and 50 female, aged 19–57) recruited on Prolific to perform the task described in Fig. 1b. All participants provided informed consent before commencing the experiment. Subjects were asked to complete six ‘guided’ episodes where the optimal path was shown explicitly, followed by 40 nonguided episodes and 12 guided episodes. The task can be found online. During data collection, a subject was deemed ‘disengaged’ and the trial was repeated if one of three conditions were met: (1) the same key was pressed five times in a row; (2) the same key pair was pressed four times in a row; or (3) no key was pressed for 7 s. Participants were paid a fixed rate of US $3 plus a performance-dependent bonus of US $0.002 for each completed trial across both guided and nonguided episodes. The experiment took approximately 22 minutes to complete and the average pay across participants was US $10.5 per hour including the performance bonus. For the experiment without periodic boundaries (Extended Data Fig. 1), we collected data from 49 human participants (25 male and 24 female). The experiment was performed as described above, with the only difference being that we used mazes where participants could not move through the boundaries.

The data from six participants with a mean response time greater than 690 ms during the guided episodes were excluded to avoid including participants who were not sufficiently engaged with the task. For the guided episodes, only the last 10 episodes were used for further analyses. For the nonguided episodes, we discarded the first two episodes and used the last 38 episodes. This was done to give participants two episodes to get used to the task for each of the two conditions, and the first set of guided episodes was intended as an instruction in how to perform the task.

#### Performance as a function of trial number

We considered all episodes where the humans or RL agents completed at least four trials, evaluating the RL agents across 50,000 episodes. We then computed the average across these episodes of the number of steps to goal as a function of trial number separately for all subjects. Figure 2a illustrates the mean and s.e.m. across subjects (human participants or RL agents). The optimal value during the exploitation phase was computed by using dynamic programming to find the shortest path between each possible starting location and the goal location, averaged across all environments seen by the RL agent. To compute the exploration baseline, a brute-force search was used to identify the path that explored the full environment as quickly as possible. The optimal exploration performance was then computed as the expected time to first reward under this policy, averaged over all possible goal locations.

#### Estimation of thinking times

In broad strokes, we assumed that, for each action, the response time *t*_r_ is the sum of a thinking time *t*_t_ and some perception–action delay *t*_d_, both subject to independent variability:

$${t}_{{{{\rm{r}}}}}={t}_{{{{\rm{t}}}}}+{t}_{{{{\rm{d}}}}}\quad {{{\rm{with}}}}\quad {t}_{{{{\rm{t}}}}} \sim {p}_{{{{\rm{t}}}}}\quad {{{\rm{and}}}}\quad {t}_{{{{\rm{d}}}}} \sim {p}_{{{{\rm{d}}}}}.$$

Here, {*t*_r_, *t*_t_, *t*_d_} ≥ 0 because elapsed time cannot be negative. We assumed that the prior distribution over perception–action delays, *p*_d_, was identical during guided and nonguided trials. For each subject, we obtained a good model of *p*_d_ (see below) by considering the distribution of response times measured during guided trials. This was possible because guided trials involved no thinking by definition, such that *t*_d_ ≡ *t*_r_ was directly observed. Finally, for any nonguided trial with observed response *t*_r_, we formed a point estimate of the thinking time by computing the mean of the posterior *p*(*t*_t_∣*t*_r_):

$${\hat{t}}_{{{{\rm{t}}}}| {t}_{{{{\rm{r}}}}}}={{\mathbb{E}}}_{\rm{p}({t}_{{{{\rm{t}}}}}| {t}_{{{{\rm{r}}}}})}[{t}_{{{{\rm{t}}}}}].$$

In more detail, we took *p*_t_ during nonguided trials to be uniform between 0 and 7 s—the maximum response time allowed, beyond which subjects were considered disengaged, and the trial was discarded and reset. For *p*_d_(*t*_d_), we assumed a shifted log-normal distribution,

$${p}_{{{{\rm{d}}}}}({t}_{{{{\rm{d}}}}};\mu ,\sigma ,\delta )=\left\{\begin{array}{cc}\frac{1}{({t}_{{{{\rm{d}}}}}-\delta )\sigma \sqrt{2\pi }}\exp \left[-\frac{{(\log ({t}_{{{{\rm{d}}}}}-\delta )-\mu )}^{2}}{2{\sigma }^{2}}\right]&{{{\rm{if}}}}\,{t}_{\rm{d}} > \delta \\ 0&{{{\rm{otherwise}}}}\end{array}\right.$$

where parameters *μ*, *σ* and *δ* were obtained from a maximum-likelihood estimation based on the collection of response times *t*_r_ ≡ *t*_d_ observed during guided trials. For a given *δ*, the maximum-likelihood values of *μ* and *σ* are simply given by the mean and s.d. of the logarithm of the shifted observations. Thus, to fit this shifted log-normal model, we performed a grid search over $\delta \in [0,\min ({t}_{r}^{{{{\rm{guided}}}}})-1]$ at 1-ms resolution and selected the value under which the optimal (*μ*, *σ*) gave the largest likelihood. This range of *δ* was chosen to ensure that (1) only positive values of ${t}_{r}^{{{{\rm{guided}}}}}$ had positive probability and (2) all observed ${t}_{r}^{{{{\rm{guided}}}}}$ had nonzero probability. We then retained the optimal *μ*, *σ* and *δ* to define the prior over *p*_d_(*t*_*d*_) on nonguided trials for each subject.

According to Bayes’ rule, the posterior is proportional to

$$p({t}_{{{{\rm{t}}}}}| {t}_{{{{\rm{r}}}}})\propto p({t}_{{{{\rm{r}}}}}| {t}_{{{{\rm{t}}}}})p({t}_{{{{\rm{t}}}}})$$

where

$$p({t}_{{{{\rm{r}}}}}| {t}_{{{{\rm{t}}}}})=\int\nolimits_{0}^{\infty }{\rm{d}}{t}_{{{{\rm{d}}}}}\,{p}_{{{{\rm{d}}}}}({t}_{{{{\rm{d}}}}})\,p({t}_{{{{\rm{r}}}}}| {t}_{{{{\rm{t}}}}},{t}_{{{{\rm{d}}}}})$$

$$=\int\nolimits_{0}^{\infty }{\rm{d}}{t}_{{{{\rm{d}}}}}\,{p}_{{{{\rm{d}}}}}({t}_{{{{\rm{d}}}}})\,\delta ({t}_{{{{\rm{d}}}}}-({t}_{{{{\rm{r}}}}}-{t}_{{{{\rm{t}}}}}))$$

$$={p}_{{{{\rm{d}}}}}({t}_{{{{\rm{r}}}}}-{t}_{{{{\rm{t}}}}})$$

Therefore, the posterior is given by

$$p({t}_{{{{\rm{t}}}}}| {t}_{{{{\rm{r}}}}})\propto \left\{\begin{array}{ll}{p}_{{{{\rm{d}}}}}({t}_{{{{\rm{r}}}}}-{t}_{{{{\rm{t}}}}})&{{{\rm{if}}}}\,{t}_{{{{\rm{t}}}}} > 0\\ 0&{{{\rm{otherwise}}}},\end{array}\right.$$

resulting in the following posterior mean:

$${\hat{t}}_{{{{\rm{t}}}}| {t}_{{{{\rm{r}}}}}}:= {{\mathbb{E}}}_{p({t}_{{{{\rm{t}}}}}| {t}_{{{{\rm{r}}}}})}[{t}_{{{{\rm{t}}}}}]={t}_{{{{\rm{r}}}}}-\int\nolimits_{\delta }^{{t}_{{{{\rm{r}}}}}}\,{t}_{{{{\rm{d}}}}}\,{p}_{{{{\rm{d}}}}}({t}_{{{{\rm{d}}}}}| {t}_{{{{\rm{d}}}}} < {t}_{{{{\rm{r}}}}};\mu ,\sigma ,\delta )\,{\rm{d}}{t}_{{{{\rm{d}}}}}.$$

Here, *p*_d_(*t*_d_∣*t*_d_ < *t*_r_) denotes *p*_d_(*t*_d_) renormalized over the interval *t*_d_ < *t*_r_ and the condition (*t*_d_ < *t*_r_) is equivalent to (*t*_t_ > 0). We note that the integral runs from *δ* to *t*_r_ because *p*_d_(*t*_d_) = 0 for *t*_d_ < *δ*. Because *δ* simply shifts the distribution over *t*_d_, we can rewrite this as

$${\hat{t}}_{{{{\rm{t}}}}| {t}_{{{{\rm{r}}}}}}={t}_{{{{\rm{r}}}}}-\delta -\int\nolimits_{0}^{{t}_{{{{\rm{r}}}}}-\delta }\,x\,{p}_{{{{\rm{d}}}}}(x| x < {t}_{{{{\rm{r}}}}}-\delta ;\mu ,\sigma ,\delta =0)\,{\rm{d}}x.$$

This is useful because the conditional expectation of a log-normally distributed random variable with *δ* = 0 is given in closed form by

$${{\mathbb{E}}}_{\mu ,\sigma }[x| x < k]=\int\nolimits_{0}^{k}\,x\,p(x| x < k;\mu ,\sigma ,\delta =0)\,{\rm{d}}x$$

$$=\exp [\mu +0.5{\sigma }^{2}]\frac{{{\Phi }}\left(\frac{\log (k)-\mu -{\sigma }^{2}}{\sigma }\right)}{{{\Phi }}\left(\frac{\log (k)-\mu }{\sigma }\right)},$$

where Φ( ⋅ ) is the cumulative density function of the standard Gaussian, ${{{\mathcal{N}}}}(0,1)$. This allows us to compute the posterior mean thinking time for an observed response time *t*_r_ in closed form as

$${\hat{t}}_{t| {t}_{{{{\rm{r}}}}}}={t}_{{{{\rm{r}}}}}-\delta -{{\mathbb{E}}}_{\mu ,\sigma }[x| x < {t}_{{{{\rm{r}}}}}-\delta ].$$

We note that the support of *p*_d_(*t*_d_∣*t*_d_ < *t*_r_; *μ*, *σ*, *δ*) is *t*_d_ ∈ [*δ*, *t*_r_]. For 0.6% of the nonguided decisions, the value of *t*_r_ was lower than the estimated *δ* for the corresponding participant, in which case *p*(*t*_t_∣*t*_r_) was undefined. In such cases, we defined the thinking time to be ${\hat{t}}_{{{{\rm{t}}}}| {t}_{{{{\rm{r}}}}}}=0$ because the response time was shorter than our estimated minimum perception–action delay. A necessary (but not sufficient) condition for *t*_r_ < *δ* is that *t*_r_ is smaller than the smallest response time in the guided trials.

The whole procedure of fitting and inference described above was repeated separately for actions that immediately followed a teleportation step (that is, the first action in each trial) and for all other actions. This is because we expected the first action in each trial to be associated with an additional perceptual delay compared to actions that followed a predictable transition.

While this approach dissociates thinking from other forms of sensorimotor processing to some extent, the thinking times reported in this work still only represent a best estimate given the available data and we use thinking to refer to any internal computational process guiding decision-making. This does not necessarily imply a conscious process that we can introspect because decision-making occurs on a fast timescale of hundreds of milliseconds.

All results were qualitatively similar using other methods for estimating thinking time, including (1) a log-normal prior over *t*_d_ with no shift (*δ* = 0); (2) using the posterior mode instead of the posterior mean; (3) estimating a constant *t*_d_ from the guided trials; and (4) estimating a constant *t*_d_ as the 0.1 or 0.25 quantile of *t*_r_ from the nonguided trials.

#### Thinking times in different situations

To investigate how the thinking time varied in different situations, we considered only exploitation trials and computed for every action (1) the minimum distance to the goal at the beginning of the corresponding trial and (2) what action number this was within the trial. We then computed the mean thinking time as a function of action number separately for each initial distance to goal. This analysis was repeated across experimental subjects and results were reported as the mean and s.e.m. across subjects.

We repeated this analysis for the RL agents, where thinking time was now defined on the basis of the average number of rollouts performed, conditioned on action within trial and initial distance to goal.

#### Comparison of human and model thinking times

For each subject and each RL agent, we clamped the trajectory of the agent to that taken by the subject (that is, we used the human actions instead of sampling from the policy). After taking an action, we recorded *π*(rollout) under the model on the first time step of the new state for comparison to human thinking times. We then sampled a rollout with probability *π*(rollout) and took an action (identical to the next human action) with probability 1 − *π*(rollout), repeating this process until the next state was reached. Finally, we computed the average *π*(rollout) across 20 iterations of each RL agent for comparison to the human thinking time in each state. Figure 2e shows the human thinking time as a function of *π*(rollout), with the bars and error bars illustrating the mean and s.e.m. in each bin. For this analysis, data were aggregated across all participants. Results were similar if we compared human thinking times with the average number of rollouts performed rather than the initial *π*(rollout).

In Fig. 2f, we computed the correlation between thinking time and various regressors on a participant-by-participant basis and reported the result as the mean and s.e.m. across participants (*n* = 94). For the residual correlation, we first computed the mean thinking time for each momentary distance to goal for each participant and the corresponding mean *π*(rollout) for the RL agents. We then subtracted the appropriate mean values from the thinking times (for human participants) and *π*(rollout) (for RL agents). In other words, we subtracted the average across all situations where the momentary position was five steps from the goal from each of the individual data points with a momentary distance of five steps from the goal, with a similar approach for all other distances. Finally, we computed the correlation between the residual *π*(rollout) and the residual thinking times. This analysis was repeated across all participants and the result was reported as the mean and s.e.m. across participants. Note that all measures of the distance to goal refer to the shortest path to goal rather than the number of steps actually taken by the participant to reach the goal.

### Analysis of hippocampal replays

For our analyses of hippocampal replays in rats, we used data recently recorded by Widloski and Foster^7^. This dataset consisted of a total of 37 sessions from three rats (*n* = 17, 12 and 8 sessions for each rat) as they performed a dynamic maze task. This task was carried out in a square arena with nine putative reward locations. In each session, six walls were placed in the arena and a single reward location was randomly selected as the home well. The task involved alternating between moving to this home well and a randomly selected away well. Importantly, a delay of 5–15 s was imposed between the animal leaving the previous rewarded well before the reward (chocolate milk) became available at the next rewarded well. On the away trials, the emergence of the reward was also accompanied by a visual cue at the rewarded well, informing the animal that this was the reward location. We considered only replays at the previous well before this visual cue and the reward became available. In a given session, the animals generally performed around 80 trials (40 home trials and 40 away trials; Extended Data Fig. 8). For further task details, refer to Widloski and Foster^7^.

For our analyses, we included only trials that lasted less than 40 s. We did this to discard time periods where the animals were not engaged with the task. Additionally, we discarded the first home trial of each session, where the home location was unknown, because we wanted to compare the hippocampal replays with model rollouts during the exploitation phase of the maze task. For all analyses, we discretized the environment into a 5 × 5 grid (the 3 × 3 grid of wells and an additional square of states around these) to facilitate more direct comparisons with our human and RNN task. Following Widloski and Foster^7^, we defined ‘movement epochs’ as times where the animal had a velocity greater than 2 cm s^−1^ and ‘stationary epochs’ as times there the animal had a velocity less than 2 cm s^−1^.

#### Replay detection

To detect replays, we followed Widloski and Foster^7^ and fitted a Bayesian decoder to neural activity as a function of position during movement epochs in each session, assuming Poisson noise statistics and considering only neurons with an average firing rate of at least 0.1 Hz over the course of the session. This decoder was trained on a rolling window of neural activity spanning 75 ms and sampled at 5-ms intervals^7^. We then detected replays during stationary epochs by classifying each momentary hippocampal state as the maximum-likelihood state under the Bayesian decoder, again using neural activity in 75-ms windows at 5-ms intervals. Forward replays were defined as sequences of states that included two consecutive transitions to an adjacent state (that is, a temporally and spatially contiguous sequence of three or more states) and originated at the true animal location. For all animals, we analyzed only replays where the animal was at the previous reward location before it initiated the new trial (refer to Widloski and Foster^7^). To increase noise robustness, we allowed for short ‘lapses’ in a replay, defined as periods with a duration less than or equal to 20 ms, where the decoded location moved to a distant location before returning to the previously decoded location. These lapses were ignored for downstream analyses.

#### Wall avoidance

To compute the wall avoidance of replays (Fig. 4b), we calculated the fraction of state transitions that passed through a wall. This was done across all replays preceding a home trial (that is, when the animal knew the next goal). As a control, we computed the same quantity averaged over seven control conditions, which corresponded to the remaining nonidentical rotations and reflections of the walls from the corresponding session. We repeated this analysis for all sessions and reported the results in Fig. 4 as the mean and s.e.m. across sessions. To test for significance, we randomly permuted the ‘true’ and ‘control’ labels independently for each session and computed the fraction of permutations (out of 10,000), where the difference between ‘control’ and ‘true’ was larger than the experimentally observed value. This analysis was also repeated in the RL agent, where the control value was computed with respect to 50,000 other wall configurations sampled from the maze-generating algorithm (Algorithm 1).

#### Reward enrichment

To compute the reward enrichment in hippocampal replays (Fig. 4c), we computed the fraction of all replays preceding a home trial that passed through the reward location. As a control, we repeated this analysis for the remaining seven locations that were neither the reward location nor the current agent location (for each replay). Control values were reported as the average across these seven control locations across all replays. This analysis was repeated for all sessions. While this can lead to systematic differences between true and control values in individual trials depending on how close the true reward location is to the current animal locations, the distance to goal will be the same in expectation between the true reward location and the control locations. This is also why we did not see an effect for the away trials in Extended Data Fig. 9c.

To test for significance, we randomly permuted the ‘goal’ and ‘control’ labels independently for each session. Here, the goal and control values permuted were those computed by averaging across all trials and control locations in the session (that is, we randomly swapped the 37 pairs of data points shown in gray in Fig. 4c). We then computed the fraction of permutations (out of 10,000) where the difference between goal and control was larger than the experimentally observed value after averaging across sessions.

This analysis was also repeated in the RL agent, where the control value was computed across the 14 locations that were not the current agent location or the true goal.

#### Behavior by replay type

To investigate how the animal behavior depended on the type of replay (Fig. 4d), we analyzed home trials and away trials separately. We constructed a list of all the ‘first’ replayed actions ${\hat{a}}_{1}$, defined as the cardinal direction corresponding to the first state transition in each replay. We then constructed a corresponding list of the first physical action following the replay, corresponding to the cardinal direction of the first physical state transition after the replay. Finally, we computed the overlap between these two vectors to arrive at the probability of ‘following’ a replay. This overlap was computed separately for successful and unsuccessful replays, where successful replays were defined as those that reached the goal without passing through a wall. For the unsuccessful replays, we considered the seven remaining locations that were not the current animal location or current goal. We then computed the average overlap under the assumption that each of these locations was the goal, while discarding replays that were successful for the true goal. The reason for not considering replays that were successful for the true goal in the unsuccessful setting is because we were primarily interested in the distinction between replays that were successful versus unsuccessful to the true goal and, therefore, wanted disjoint sets of replays in these two analyses. However, we did this while considering whether replays were successful toward a control location to better match the spatiotemporal statistics of replays in the two categories. The analysis was performed independently across all sessions and results were reported as the mean and s.e.m. across sessions. To test for significance, we randomly permuted the successful and unsuccessful labels independently for each session and computed the fraction of permutations (out of 10,000) where the difference between successful and unsuccessful replays was larger than the experimentally observed value.

To confirm that our results were not biased by the choice to exclude replays that were successful to the true goal location from our set of unsuccessful replays, we performed an additional control analysis, where the control replays were the full set of replays that were successful toward a randomly sampled control location, concatenated across control locations. In this case, the control value for the home trials was $P({a}_{1}={\hat{a}}_{1})=0.433$ instead of $P({a}_{1}={\hat{a}}_{1})=0.403$ with the disjoint set of unsuccessful replays used in the main text. This is still significantly smaller than the value of $P({a}_{1}={\hat{a}}_{1})=0.622$ for the true goal location, with our permutation test in both cases yielding *P* < 0.001 for the home trials and no significant effect for the away trials.

This analysis was also repeated for the RL agent, where we considered all exploitation trials together because they were not divided into home or away trials. In this case, the control was computed with respect to all 14 locations that were not the current location or current goal location.

#### Effect of consecutive replays

To compute how the probability of a replay being successful depended on replay number (Fig. 4e), we considered all trials where an animal performed at least three replays. We then computed a binary vector indicating whether each replay was successful. From this vector, we subtracted the expected success frequency from a linear model predicting success from (1) the time since arriving at the current well and (2) the time until departing the current well. We did this to account for any effect of time that was separate from the effect of replay number because such an effect was previously reported by Ólafsdóttir et al.^39^. However, this work also notes that many of what they denoted as disengaged replays were nonlocal and would automatically be filtered out by our focus on local replays. When fitting this linear model, we capped all time differences at a maximum value of ∣Δ*t*∣ = 15 s to avoid the analysis being dominated by outliers and because Ólafsdóttir et al.^39^ observed an effect for time differences only in this range. Our results were not sensitive to altering or removing this threshold. We then conditioned on replay number and computed the probability of success (after regressing out time) as a function of replay number. Finally, we repeated this analysis for all seven control locations for each replay and divided the true values by control values defined as the average across replays of the average across control locations. A separate correction factor was subtracted from these control locations, which was computed by fitting a linear model to predict the average probability of successfully reaching a control location as a function of the predictors described above. The normalization by control locations was performed to account for changes in replay statistics that might affect the results, such as systematically increasing or decreasing replay durations with replay number. To compute the statistical significance of the increase in goal over-representation, we also performed this analysis after independently permuting the order of the replays in each trial to break any temporal structure. This permutation was performed after regressing out the effect of time. We repeated this analysis across 10,000 independent permutations and computed statistical significance as the number of permutations for which the increase in over-representation was greater than or equal to the experimental value.

For the corresponding analysis in the RL agents, we did not regress out time because there is no separability between time and replay number. Additionally, the RL agent cannot alter its policy in the absence of explicit network updates, which are tied to either a rollout or an action in our model. As noted in the main text, an increase in the probability of success with replay number in the RL agent could also arise from the fact that performing further replays is less likely after a successful replay than after an unsuccessful replay (Extended Data Fig. 10). We, therefore, performed the analysis of consecutive replays in the RL agent in a ‘cross-validated’ manner at the level of the policy. In other words, every time the agent performed a rollout, we drew two samples from the rollout generation process. The first of these samples was used as normal by the agent to update *h*_k_ and drive future behavior. The second sample was not used by the agent but was instead used to compute the ‘success frequency’ for our analyses. This was done to break the correlation between the choice of performing a replay and the assessment of how good the policy was, which allowed us to compute an unbiased estimate of the quality of the policy as a function of replay number. As mentioned in the main text, such an analysis was not possible for the biological data. However, because the biological task was not a reaction time task, we expect less of a causal effect of replay success on the number of replays. Additionally, as noted in the text, if some of the effect in the biological data is in fact driven by a decreased propensity for further replays after a successful replay, that is in itself supporting evidence for a theory of replays as a form of planning.

### Reporting summary

Further information on research design is available in the Nature Portfolio Reporting Summary linked to this article.

## Online content

Any methods, additional references, Nature Portfolio reporting summaries, source data, extended data, supplementary information, acknowledgements, peer review information; details of author contributions and competing interests; and statements of data and code availability are available at 10.1038/s41593-024-01675-7.

### Supplementary information

Supplementary Information Supplementary Notes 1 and 2.

Reporting Summary

### Source data

Source Data Fig. 1 Source data for Figs. 2–4 and Extended Data Figs. 1–10.

## Data Availability

Human behavioral data are available on GitHub (https://github.com/KrisJensen/planning_code). The rodent data are available upon request from Widloski and Foster^7^, who recorded the data. Source data are provided with this paper.

## Code availability

Code for model training and all analyses is available on GitHub.
