# Supplementary material for: A recurrent network model of planning explains hippocampal replay and human behavior
Source: Nat Neurosci. 2024 Jun 7;27(7):1340–8. doi: 10.1038/s41593-024-01675-7 (PMC11239510; doi:10.1038/s41593-024-01675-7)
Supplement: Supplementary file 1 — Supplementary Notes 1 and 2. [file 41593_2024_1675_MOESM1_ESM.pdf]

nature neuroscience

Supplementary information

https://doi.org/10.1038/s41593-024-01675-7

A recurrent network model of planning explains hippocampal replay and human behavior

In the format provided by the authors and unedited

Contents

1 Relationship between rollouts and policy gradients 1

2 Discussion of experimental and architectural choices 3

Supplementary note 1: Relationship between rollouts and policy gradients

In the main text, we saw that both biological and artificial agents appear to use policy rollouts to improve behavior. Since all weights of the RL agent are fixed at test time, the only way to achieve this is through improvement of the hidden state of the agent. Such an optimization process requires the agent to approximate the *gradient* of the expected reward with respect to its hidden state, using information from the rollout. To understand how this is possible, we recall that optimization of the weights of the network uses precisely such policy rollouts in a policy gradient algorithm to approximate the gradient of the expected reward with respect to the network parameters. These algorithms consider putative on-policy action sequences τ and apply *parameter updates* that cause $p(\tau)$ to increase under the agent's policy if τ led to more reward than expected, and to decrease otherwise [1]. In our trained agent, adaptation to each new maze does not involve modifications of the fixed network parameters but instead occurs through changes to the *hidden state* $\boldsymbol{h}_k$. The performance improvements resulting from policy rollouts (Figure 3a; Figure 4e) can therefore be achieved through iterative modifications of $\boldsymbol{h}_k$ that approximate policy gradient ascent on the expected future reward in the episode as a function of $\boldsymbol{h}_k$ (Figure S1a). Importantly, while the optimization at the outer loop was hardcoded, this 'inner' policy gradient algorithm, operating on the basis of imagined experience, has to be meta-learned by the agent itself over the course of many episodes of the navigation task.

To verify that the algorithm implemented by the agent approximates such single-sample policy gradient estimates, we considered each rollout performed by the RL agent and computed both (i) the actual hidden state update performed on the basis of this rollout, and (ii) the expected hidden state update computed by applying the policy gradient algorithm to the same rollout (Figure S1b; Methods). The policy gradient algorithm specifies that rollouts should change $\boldsymbol{h}_k$ in a way that increases $p(\tau = \hat{\tau})$ if the rollout is better than some baseline and decreases $p(\tau = \hat{\tau})$ otherwise. Since we do not know the baseline, we performed our analysis by taking the *derivative* of the hidden state change with respect to the expected reward from physically following $\hat{\tau}$, $R_{\hat{\tau}}$, which is independent of the baseline (Methods). This allowed us to define (i) a quantity $\boldsymbol{\alpha}^{\text{PG}} := \frac{\partial \Delta \boldsymbol{h}^{\text{PG}}}{\partial R_{\hat{\tau}}}$ that predicts how the hidden state *should* change as a function of $R_{\hat{\tau}}$ in the policy gradient formulation, and (ii) the corresponding quantity $\boldsymbol{\alpha}^{\text{RNN}} := \frac{\partial \Delta \boldsymbol{h}^{\text{RNN}}}{\partial R_{\hat{\tau}}}$ that indicates how the hidden state *actually* changed as a function of the content of the rollout. If the agent performs approximate policy gradient ascent in hidden state space, $\boldsymbol{\alpha}^{\text{RNN}}$ should be aligned with $\boldsymbol{\alpha}^{\text{PG}}$.

We began by considering the effect of the first action in the rollout, $\hat{a}_1$, on the hidden state of the agent. We did this by querying the alignment between (i) $\boldsymbol{\alpha}^{\text{RNN}}$ computed across rollouts from 1,000 episodes, and (ii) $\boldsymbol{\alpha}_1^{\text{PG}}$ computed from the same rollouts when considering only the probability of executing $\hat{a}_1$. To visualize this alignment, we performed PCA on $\{\boldsymbol{\alpha}_1^{\text{PG}}\}$ from all rollouts and projected both $\boldsymbol{\alpha}_1^{\text{PG}}$ and $\boldsymbol{\alpha}^{\text{RNN}}$ into this low-dimensional subspace. We then computed the average of each of these two quantities for each simulated action $\hat{a}_1 \in \{\text{left, right, up, down}\}$. The average value of $\boldsymbol{\alpha}^{\text{RNN}}$ was strongly aligned with the average value of $\boldsymbol{\alpha}_1^{\text{PG}}$ for each action (Figure S1c), consistent with the policy gradient algorithm. This implies that $R_{\hat{\tau}}$ has *different* effects on the policy depending on the replayed trajectory $\hat{\tau}$. In other words, the spatial content of the rollout dynamically modulates the way in which the reward signal from the rollout affects the hidden state and policy of the agent.

To quantify the overlap between $\boldsymbol{\alpha}^{\text{RNN}}$ and $\boldsymbol{\alpha}_1^{\text{PG}}$ on a rollout-by-rollout basis, we computed the average cosine similarity d between $\boldsymbol{\alpha}^{\text{RNN}}$ and $\boldsymbol{\alpha}_1^{\text{PG}}$ across all rollouts. This overlap was substantially larger than zero ($d = 0.49 \pm 0.02$ mean $\pm$ sem; Figure S1d, left). When instead computing the overlap with $\boldsymbol{\alpha}_{\text{ctrl}}^{\text{RNN}}$ after changing the feedback input to falsely inform the agent that it simulated a different action $\hat{a}_{1,\text{ctrl}} \neq \hat{a}_1$, the similarity was $d = -0.15 \pm 0.01$. This confirms that $\boldsymbol{h}_k$ is optimized by incorporating the specific feedback input obtained from the rollout, and the negative sign reflects anti-correlations due to the policy being a normalized distribution over actions. For these analyses, we only considered the first simulated action $\hat{a}_1$. When instead querying the effect of the rollout on subsequent actions in $\hat{\tau}$, we found that the feedback input was also propagated through the network dynamics to these later actions, although with a weaker alignment than for the first action (Figure S1d, right). This weaker effect for the second action could arise because the agent has the capacity to re-plan at each iteration, which is not taken into account in the rollouts, or it could simply reflect additional noise resulting from the network dynamics.

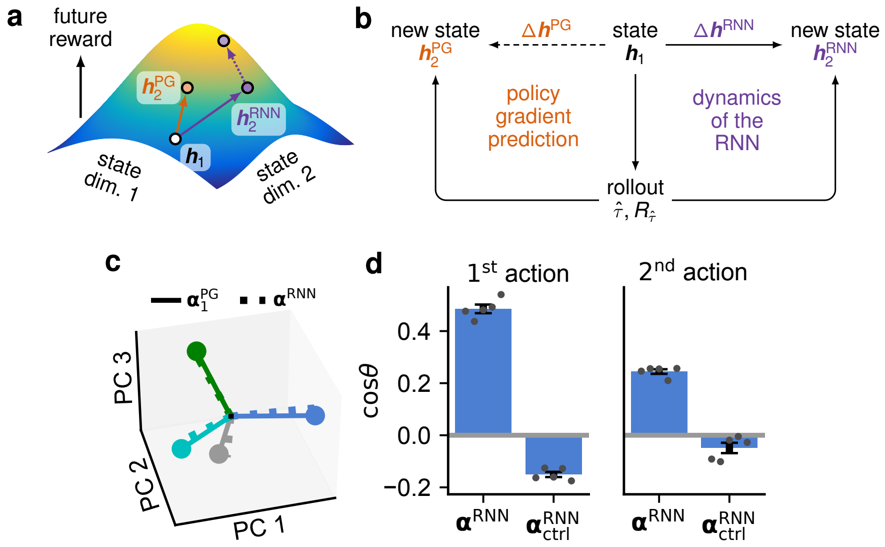

Figure S1: **Rollouts implement a hidden state optimization.** **(a)** The hidden state $\boldsymbol{h}_k$ of the RNN induces a policy with an expected future reward for the current episode, setting the 'initial state' of a dynamical system consisting of both the agent itself and the environment. Rollouts can improve performance by shifting $\boldsymbol{h}$ to a region of state space with higher reward for a given environment and agent location ($\boldsymbol{h}_1 \rightarrow \boldsymbol{h}_2^{\text{RNN}}$). The policy gradient algorithm uses samples from the policy to estimate the direction of steepest ascent of the expected reward as a function of $\boldsymbol{h}_k$ ($\boldsymbol{h}_1 \rightarrow \boldsymbol{h}_2^{\text{PG}}$). **(b)** We compare this theoretical hidden state update $\Delta\boldsymbol{h}^{\text{PG}} := \boldsymbol{h}_2^{\text{PG}} - \boldsymbol{h}_1$ to the empirical hidden state update $\Delta\boldsymbol{h}^{\text{RNN}} := \boldsymbol{h}_2^{\text{RNN}} - \boldsymbol{h}_1$ actually performed by the network dynamics on the basis of a rollout $\hat{\tau}$ and its associated reward $R_{\hat{\tau}}$. **(c)** A latent space was defined by performing PCA on $\boldsymbol{\alpha}_1^{\text{PG}}$ – the effect of $R_{\hat{\tau}}$ on $\boldsymbol{h}_k$ under the policy gradient algorithm. Solid lines and circles indicate the normalized average $\boldsymbol{\alpha}_1^{\text{PG}}$ for each of the four possible simulated actions ($\hat{a}_1$; colors). Dashed lines indicate the normalized average values of $\boldsymbol{\alpha}^{\text{RNN}}$ for the corresponding actions, which are aligned with $\boldsymbol{\alpha}_1^{\text{PG}}$ in accordance with the theory. The first 3 PCs capture 100% of the variance in $\boldsymbol{\alpha}_1^{\text{PG}}$, since the policy is normalized and therefore only has three degrees of freedom. **(d)** Average cosine similarity between $\boldsymbol{\alpha}^{\text{RNN}}$ and $\boldsymbol{\alpha}_1^{\text{PG}}$, quantified in the space spanned by the top 3 PCs of $\boldsymbol{\alpha}_1^{\text{PG}}$. $\boldsymbol{\alpha}^{\text{RNN}}$ was computed using the true input, while $\boldsymbol{\alpha}_{\text{ctrl}}^{\text{RNN}}$ was computed after altering the feedback from the rollout to falsely inform the agent that it had simulated a different action $\hat{a}_{1,\text{ctrl}} \neq \hat{a}_1$. Left panel considers the effect of $R_{\hat{\tau}}$ on the first action ($\boldsymbol{\alpha}_1^{\text{PG}}$) and right panel considers the effect of $R_{\hat{\tau}}$ on the second action ($\boldsymbol{\alpha}_2^{\text{PG}}$). Error bars indicate standard error across five RL agents (gray dots).

Supplementary note 2: Discussion of experimental and architectural choices

In this note, we discuss some of the many architectural and modeling choices that went into our work. As is the case for much work in modern computational neuroscience, the space of models was vast – and larger than we could feasibly explore fully in a single paper. In what follows, we hope to provide some additional motivation for the choices that were made in the main paper and to provide additional intuition for the importance and effect of various architectural choices and hyperparameters in our work. This note is also unlikely to be exhaustive, but we hope that it will be useful both for the reader hoping to gain a deeper understanding of our work, and for those looking to draw inspiration from it in their own computational models.

Network size

The size of the network used in our work is of some importance. We show in Extended Data Figure 4 that our key results hold across a range of different network sizes. However, as the network becomes larger, its model-free 'base policy' also becomes better – to the point where rollouts become less and less useful as there is less room for improvement from the base policy. Indeed, in the limit of an infinitely large network trained on an infinitely large dataset, we expect a perfect base policy and no rollouts. On the contrary, if the network gets too small, it is unlikely to be able to learn how to use the rollouts for policy improvement, and we again expect rollouts to be less useful. In both limits, we also expect the notions of 'large' and 'small' to depend on the complexity of the task in question. While it may seem like a limitation of this work that the results 'only' hold for certain choices of task and network size, we believe that this is consistent with how humans and other animals operate. In particular, if we are given a particularly easy task, like navigation to a location 100 meters down the road, we are unlikely to spend any time thinking. Similarly, if we are given a seemingly impossible task, like solving a very long and complicated equation, we may simply make a guess without taking the time to think through every step. These notions of 'very easy' and 'very difficult' are likely to differ across animals with different cognitive capabilities.

For the task considered in this study, we found substantial use of rollouts across a range of network sizes between 40 to 140, and we found that the frequency of rollouts tended to decrease with network size (Extended Data Figure 2). We did not test any networks larger than 140 units due to computational constraints associated with the training of large networks. Networks smaller than 40 units also exhibited reduced rollout frequencies, which we hypothesize is because they do not have the capacity to learn both how to solve the task and how to take advantage of the rollout machinery. The exact range of sizes for which our results hold will also depend on the type of network used, with LSTMs likely to be similar to the GRUs used in this work, and vanilla RNNs probably needing larger networks for comparable performance.

Planning horizon

In this work, we assumed a constant maximum planning horizon of $L = 8$ steps and showed in Extended Data Figure 4 that our key results are robust to changes to this hyperparameter between 4 and 12 (we did not test values outside this range). We chose a value of 8 for the main paper since it seemed like a reasonable planning depth in our fairly simple task with a relatively small action space, and it is comparable to the planning depth estimated in other simple games [2]. It is worth noting that some aspects of our results do change with planning depth. In particular, the change in policy for successful and unsuccessful rollouts is dependent on L, such that longer planning horizons lead to smaller average policy changes for successful rollouts and larger changes for unsuccessful rollouts. In the language of policy gradients, we expect that this is because the 'baseline' implicitly used by the network in its state update is related to the average success of a rollout. In other words, if almost all rollouts are successful (because L is sufficiently large), little is learned by observing that a rollout is successful, and the policy should not change much on the basis of this information. It is possible that there will be larger average policy changes in this setting if we instead condition on how late in the rollout the reward was found, which contains more information about the 'goodness' of the replayed trajectory. It would also be possible to make the planning horizon *variable* and let the agent itself choose its planning depth. This could be done in two different ways, namely by (i) making the agent decide up front how long a trajectory it wants to simulate, or (ii) letting the agent decide in closed-loop by iteratively returning a partial plan and deciding whether to continue planning or terminate and take a physical action. We opted for the simple fixed length solution since it has a smaller action space and fewer network iterations, making optimization easier. However, a variable planning length model may be closer to human behavior and could be an interesting avenue for future research.

Time cost of acting and planning

Related to the discussion of planning depth, we also assumed a constant temporal opportunity cost of planning for the agent in the main text (see Extended Data Figure 6 for results with a variable temporal opportunity cost). This was done despite the rollouts having variable length depending on whether and when the goal was reached during the rollout. We did this because the agent did not know *a priori* how long the rollout would be and had no direct control over its length. In the case of hippocampal replays being contained in sharp-wave ripples (SWRs), this is consistent with an assumption that a single trajectory occurs in a single SWR, and that the inter-SWR interval is independent of the length of the replayed trajectory.

More specifically, we defined a rollout in the model to last 120 ms. This is similar to the duration of human hippocampal replays reported in the literature [3]. In contrast, a single model action was defined to take 400 ms. These values were not directly fitted to the human data, as all model hyperparameters, including the episode length and relative cost of planning and acting, were chosen before any analyses of human behavior to avoid overfitting. Instead, the relative cost of rollouts compared to actions in the model, $\beta_{\text{roll}} := \Delta t_{\text{rollout}} / \Delta t_{\text{action}} = 0.3$, was chosen such that there was regular use of rollouts in the task. The episode length $T = 50$ *actions* was chosen to facilitate training of the model. We then designed our human behavioral experiment in a way that allowed participants to take approximately the same number of actions in a given episode as the model, which motivated an episode length of $T = 20$ *seconds*. This implicitly defined the 'duration' of a model action as $\Delta t_{\text{action}} = 20$ seconds/50 actions $= 400$ ms. The duration of a rollout was then defined as $\Delta t_{\text{rollout}} = \beta_{\text{roll}} \times 400$ ms $= 120$ ms. Since we did not explicitly fit these parameters to the data, there are likely to be a range of parameter choices that lead to better fits to the human data from this particular experiment. Similarly, there is certainly a range of hyperparameters that lead to worse data fits. Indeed our goal was not to chase the lowest possible discrepancy from human response times, but rather to demonstrate the general concept that models with the ability to perform rollouts do so in similar situations to humans. This is also the reason that we focus on correlations in the paper rather than e.g. MSEs, since the model 'thinking times' can be stretched, compressed, and shifted to different extents by altering the model hyperparameters.

Policy used for planning

In our work, we assumed that the policy used within the planning loop (i.e. the policy from which actions were sampled during a rollout) was the same as the policy used for sampling actions when actually interacting with the environment. We did this both for simplicity of exposition and computation, and because we think it is likely to be a reasonable approximation to how humans plan. However, there is in theory nothing in our model that prevents the rollout policy from differing from the action policy. In this case, rollouts can still be used to estimate gradients of the future reward with respect to the hidden state, provided that the policy from which rollout actions are sampled is known. This could be done e.g. through the use of importance sampling for off-policy learning [4, 5]. Such off-policy hippocampal sequence generation has also formed the basis of other recent theories of the role of hippocampus in planning and decision making [6, 7].

In the case of sequential replays, it is plausible that previous replays directly affect future replays, e.g. in a process of exploration. In our current model, there was no option to systematically explore, and previous replays only affected future replays through their effect on the base policy. In theory, it would also be possible to more systematically explore the state space using sequential replays, and indeed we did experiment with 'rollouts' corresponding to node expansions of more advanced search algorithms, which can similarly be used to drive improved decision making. More generally, it would also be possible to optimize the rollout policy explicitly for planning by differentiating through the rollout process. This is in contrast to the present work, where the rollout policy was tied to the base policy, and rollouts were treated as part of the 'environment'. This meant that there was no propagation of gradients to allow for explicit adaptation of the policy to be better for planning.

Feedback from planning

When performing a rollout, the agent received an additional input on the subsequent timestep consisting of (i) a flattened array of the simulated actions, and (ii) a binary input indicating whether or not the rollout reached the (imagined) goal. Another reasonable choice of feedback input would be to return the sequence of *states* instead of the sequence of *actions*, or potentially to return both. Our reason for favoring the action sequence was primarily that the action space is lower dimensional (4) than the state space (16), which means that the input dimensionality is much lower than it would have been for the state sequence, assuming a one-hot encoding. This does raise the question of where this action sequence would emerge in biological circuits, given that hippocampal replays are

canonically assumed to contain spatial information. However, we consider it reasonable that this state information could be converted to information about the actions that would take you there. Instead of returning a binary input of whether the goal was reached, the rollout process could also return the output of a learned value function. We did experiment with returning both the binary 'goal' feedback and the imagined value function, and we found that the agent predominantly used the goal information in this case. We therefore chose to remove the learned value from the feedback to simplify the model. This choice is also consistent with previous work in the psychology literature suggesting that human decision making relies on binary evaluations of the success of mental samples [8]. However, we imagine that returning a learned value function would be useful in more complicated tasks with multiple or non-binary rewards.

Stochastic environments and multiple goals

For simplicity, we assumed that the environment was deterministic and that there was only a single goal. However, our model could also be extended to the setting of stochastic environments and multiple or non-binary rewards. In the case of stochastic environments, the agent would still need to simulate a *sample* from the policy. The internal world model was already trained to generate a distribution over new states, and in stochastic environments, we would want to sample from this distribution instead of using the maximum likelihood next state. Provided that the agent has learned a well-calibrated distribution over state transitions, the resulting rollout should still provide an unbiased estimate of the gradient of expected future reward with respect to the hidden state. In the case of multiple goals, it would still be possible to use the agent as-is and return a binary indicator of whether the agent reached any (or each) goal. However, as noted above, it would also be possible to return a learned value estimate instead of the binary goal information. In cases where these goals do not lead to random teleportation, it could also be useful to let the rollout continue beyond the goal. We chose not to do so in the present work, since the transition after reaching the goal was entirely unpredictable, so the simulated action sequence beyond this point would not be informative of expected reward.

Space in which to plan

We chose rollouts to occur in the space of states and observations. More specifically, the agent had to predict the upcoming state $\boldsymbol{s}_k$, and a new observation $\boldsymbol{x}_k$ was constructed automatically from $\boldsymbol{s}_k$ during the rollout. An alternative would have been to directly learn to predict $\boldsymbol{x}_k$, which we decided not to do since the majority of the input was constant within an episode. However, in more general task settings, where the environment is more variable, it might be simpler to predict the input directly. Additionally, in partially observable environments, there is a weaker correspondence between states and observations, and rollouts would require samples from the distribution over possible observations. This could either be done directly in observation space or indirectly via some inferred (or known) latent state space.

We consider it likely that humans do not plan explicitly in pixel space and instead use some form of latent planning representation. In the present work, this was also the case to some extent, since the agent input was already an abstract representation. However, in future work, it could be interesting to use a learned latent space instead. This could e.g. be done by training an autoencoder to reconstruct the state and reward information as in the VariBAD model [9]. Alternatively, planning could take place in a latent space explicitly optimized to yield good plans as in MuZero [10]. We did not experiment with any of these possibilities but believe that the results would be comparable to our present work. A major reason for our choice to implement planning in the space of state transitions is that performing high-fidelity rollouts in state space only requires the agent to learn a state transition function. As has been demonstrated in previous work, a transition function could feasibly be learned in a self-supervised manner [11], allowing agents to learn how to plan with little task-specific information. Additionally, rollouts in state space have close parallels to hippocampal replays as detailed in Figure 4.

Alternative planning algorithms

While we focused on a replay-inspired rollout algorithm as a way of 'thinking', several potential alternatives exist. For example, methods based on Monte Carlo tree search have proven powerful in the machine learning literature, where they have led to breakthroughs in the performance of reinforcement learning agents playing chess, Go, and shogi [10, 12]. However, such MCTS-based methods need to keep track of visit frequencies to a full tree of downstream nodes, which has a higher memory cost than the linear rollouts considered here. While there is in general no reason that the computational budget for MCTS cannot be adaptively allocated, most implementations also use a fixed computational budget with hundreds of node expansions [10, 12]. This is in contrast to the adaptive use of a handful

of rollouts in the agents considered in this work.

Choice of task

The task used for human behavioral experiments and RL agents differs somewhat from the task used for the hippocampal replay data. Notable differences include (i) the presence of 'away' trials in the rodent data instead of the teleportation step in the human data, (ii) the different maze sizes and wall configurations, and (iii) the presence of a forced delay between rewards in the rodent data. A natural question is thus why we did not match the human and RL task to the rodent task, which we could not change since this analysis used previously published data. There were a few major reasons for our decision to use different tasks for the humans and RL agents compared to the rodent experiments. One is that the rodent task was not a reaction time task, meaning that there was a forced delay between consecutive rewards. If we introduced a similar delay in the human task, there would be less incentive to act fast. Unfortunately, since we do not have access to intracortical recordings from the human subjects, the speed of acting is the major signal we analyse from our human participants, and it is therefore necessary with a reaction time task. Of course we could still have included away trials and used similar arenas without enforcing a delay between rewards. However, we cared mostly about the 'home' trials and therefore saw no reason to make participants spend half their time on 'away' trials. Additionally, the smaller Euclidean maze used in the rodent experiments would likely have been too simple for humans and reduced our signal to noise ratio, since there would be less time spent thinking. A simpler task and arena might similarly be simple enough that our RNNs could solve it in a fully 'model-free' manner without relying on rollouts to the same extent as in the present work. It is interesting to note that such suboptimality is a key factor of our results, but we believe that this is representative of human behavior as well, where thinking is mostly utilized in scenarios where we do not already know what to do.

Regularizing time or energy

In our RL agent, we did not incorporate any explicit energy costs for either actions or rollouts. Instead, the only unit of 'cost' was time elapsed. We did this since the only explicit incentive to be efficient in our human task was that fast decision making and good actions left more time for collecting reward. It could of course be argued that there is also some energy cost associated with taking actions in our online task, but (i) this energy cost is likely to be negligible, and (ii) if we wanted to model such energy costs in the RL agent, it would require us to introduce an additional hyperparameter to convert between 'energy' and 'time'. We considered it more interpretable and robust to only operate in the space of time, and we also believe that this is representative of many tasks encountered in our daily lives.

Biological interpretation of the RNN

As mentioned in the main text, we use 'prefrontal cortex' to refer to a broader prefrontal network consisting of PFC itself and associated areas of the basal ganglia and thalamus. This follows the work of Wang et al. [13], which suggested that this prefrontal network can be well modeled across a wide range of tasks as a recurrent meta-reinforcement learner. However, referring to the recurrent part of our RL agent as 'PFC' should be seen as a *hypothesis* rather than an *assumption* – indeed the computational and data analysis results all hold equally well if some of the functionality of our RNN is instead carried out in e.g. the hippocampal formation.

A functional argument for expecting prefrontal cortex to be important in the computations carried out by the RNN is its important role in meta-cognition [14]. There is also an extensive associated literature on the role of frontal cortex in value coding [15] and model-based behavior [16]. The combination of these functionalities makes PFC a natural candidate region in our computational model for deciding whether to plan or act as well as coordinating existing policies with new information from the planning process itself.

References

[1] Jensen, K. T. An introduction to reinforcement learning for neuroscience. *arXiv preprint arXiv:2311.07315* (2023).

[2] van Opheusden, B. *et al.* Expertise increases planning depth in human gameplay. *Nature* 1–6 (2023).

[3] Kurth-Nelson, Z., Economides, M., Dolan, R. J. & Dayan, P. Fast sequences of non-spatial state representations in humans. *Neuron* **91**, 194–204 (2016).

[4] Peshkin, L. & Shelton, C. R. Learning from scarce experience. *arXiv preprint cs/0204043* (2002).

[5] Jie, T. & Abbeel, P. On a connection between importance sampling and the likelihood ratio policy gradient. *Advances in Neural Information Processing Systems* **23** (2010).

[6] McNamee, D. C., Stachenfeld, K. L., Botvinick, M. M. & Gershman, S. J. Flexible modulation of sequence generation in the entorhinal–hippocampal system. *Nature neuroscience* **24**, 851–862 (2021).

[7] Mattar, M. G. & Daw, N. D. Prioritized memory access explains planning and hippocampal replay. *Nature neuroscience* **21**, 1609–1617 (2018).

[8] Stewart, N., Chater, N. & Brown, G. D. Decision by sampling. *Cognitive psychology* **53**, 1–26 (2006).

[9] Zintgraf, L. *et al.* VariBAD: A very good method for Bayes-adaptive deep RL via meta-learning. *arXiv preprint arXiv:1910.08348* (2019).

[10] Schrittwieser, J. *et al.* Mastering Atari, Go, chess and shogi by planning with a learned model. *Nature* **588**, 604–609 (2020).

[11] Whittington, J. C. *et al.* The Tolman-Eichenbaum machine: Unifying space and relational memory through generalization in the hippocampal formation. *Cell* **183**, 1249–1263 (2020).

[12] Silver, D. *et al.* A general reinforcement learning algorithm that masters chess, shogi, and Go through self-play. *Science* **362**, 1140–1144 (2018).

[13] Wang, J. X. *et al.* Prefrontal cortex as a meta-reinforcement learning system. *Nature neuroscience* **21**, 860–868 (2018).

[14] Botvinick, M. M. & Cohen, J. D. The computational and neural basis of cognitive control: charted territory and new frontiers. *Cognitive science* **38**, 1249–1285 (2014).

[15] Rushworth, M. F. & Behrens, T. E. Choice, uncertainty and value in prefrontal and cingulate cortex. *Nature neuroscience* **11**, 389–397 (2008).

[16] Killcross, S. & Coutureau, E. Coordination of actions and habits in the medial prefrontal cortex of rats. *Cerebral cortex* **13**, 400–408 (2003).
