# Supplementary material for: A recurrent network model of planning explains hippocampal replay and human behavior
Source: Nat Neurosci. 2024 Jun 7;27(7):1340–8. doi: 10.1038/s41593-024-01675-7 (PMC11239510; doi:10.1038/s41593-024-01675-7)
Supplement: Supplementary file 2 — Reporting Summary [file 41593_2024_1675_MOESM2_ESM.pdf]

nature portfolio

Corresponding author(s): Kristopher Torp Jensen
Last updated by author(s): Apr 18, 2024

Reporting Summary

Nature Portfolio wishes to improve the reproducibility of the work that we publish. This form provides structure for consistency and transparency in reporting. For further information on Nature Portfolio policies, see our Editorial Policies and the Editorial Policy Checklist.

Statistics

For all statistical analyses, confirm that the following items are present in the figure legend, table legend, main text, or Methods section.

n/a	Confirmed	
☐	☒	The exact sample size (*n*) for each experimental group/condition, given as a discrete number and unit of measurement
☐	☒	A statement on whether measurements were taken from distinct samples or whether the same sample was measured repeatedly
☐	☒	The statistical test(s) used AND whether they are one- or two-sided<br>*Only common tests should be described solely by name; describe more complex techniques in the Methods section.*
☐	☒	A description of all covariates tested
☐	☒	A description of any assumptions or corrections, such as tests of normality and adjustment for multiple comparisons
☐	☒	A full description of the statistical parameters including central tendency (e.g. means) or other basic estimates (e.g. regression coefficient) AND variation (e.g. standard deviation) or associated estimates of uncertainty (e.g. confidence intervals)
☐	☒	For null hypothesis testing, the test statistic (e.g. *F*, *t*, *r*) with confidence intervals, effect sizes, degrees of freedom and *P* value noted<br>*Give P values as exact values whenever suitable.*
☒	☐	For Bayesian analysis, information on the choice of priors and Markov chain Monte Carlo settings
☒	☐	For hierarchical and complex designs, identification of the appropriate level for tests and full reporting of outcomes
☐	☒	Estimates of effect sizes (e.g. Cohen's *d*, Pearson's *r*), indicating how they were calculated

Our web collection on statistics for biologists contains articles on many of the points above.

Software and code

Policy information about availability of computer code

Data collection	Human behavioral experiments were written in OCaml 5.0, with the front-end transpiled to javascript for running in the participants' browsers.
Data analysis	All models were trained in Julia version 1.7 using Flux and Zygote for automatic differentiation. All analyses of the models and human data were performed in Julia version 1.8. All analyses of hippocampal replay data were performed in Python 3.8. Code for training models and performing all analyses is available at https://github.com/KrisJensen/planning_code.

For manuscripts utilizing custom algorithms or software that are central to the research but not yet described in published literature, software must be made available to editors and reviewers. We strongly encourage code deposition in a community repository (e.g. GitHub). See the Nature Portfolio guidelines for submitting code & software for further information.

Data

Policy information about availability of data

All manuscripts must include a data availability statement. This statement should provide the following information, where applicable:

- Accession codes, unique identifiers, or web links for publicly available datasets
- A description of any restrictions on data availability
- For clinical datasets or third party data, please ensure that the statement adheres to our policy

Human behavioral data is available at https://github.com/KrisJensen/planning_code/tree/main/human_data. For the hippocampal replay data, we refer to Widloski & Foster (2022).

Human research participants

Policy information about studies involving human research participants and Sex and Gender in Research.

Reporting on sex and gender	75 male and 74 female participants were recruited for this study, as self-reported by the participants on Prolific. All analyses were performed across all participants.
Population characteristics	74 female and 75 male participants, aged 19-57.
Recruitment	Participants were recruited on Prolific and all studies were conducted online. This leads to a self-selection bias towards more tech-savvy participants, but we do not expect this to substantially affect our results since the task does not require advanced technical expertise. All participants provided informed consent prior to commencing the experiment.
Ethics oversight	UC San Diego Human Research Protection Program

Note that full information on the approval of the study protocol must also be provided in the manuscript.

Field-specific reporting

Please select the one below that is the best fit for your research. If you are not sure, read the appropriate sections before making your selection.

☐ Life sciences ☒ Behavioural & social sciences ☐ Ecological, evolutionary & environmental sciences

For a reference copy of the document with all sections, see nature.com/documents/nr-reporting-summary-flat.pdf

Behavioural & social sciences study design

All studies must disclose on these points even when the disclosure is negative.

Study description	Quantitative analyses of human behavioral data collected online using the Prolific platform.
Research sample	As we were interested in the behavior of adult humans, participants were recruited from 'all countries available' on Prolific with an age range set to 18-60 years and an approval rating of at least 95%. The final participant pool consisted of 74 female and 75 male participants, aged 19-57.
Sampling strategy	A pilot study was conducted with 10 research participants using a preliminary version of the experimental paradigm, which indicated notable but weak effects. A separate set of 100 participants were then used for the main study to increase statistical power.
Data collection	All experiments were conducted online using the Prolific platform. Experimenters were not present during data collection and did not influence or interact with participants during the experiment.
Timing	Four separate datasets were collected. Three were collected for the main study on 5th October 2022 (10 participants), 6th October 2022 (40 participants), and 14th October 2022 (50 participants). One dataset was collected for the analysis without periodic boundaries on 20th July 2023 (49 participants).
Data exclusions	The data from 6 participants with a mean response time greater than 690 ms during the guided episodes were excluded to avoid including participants who were not sufficiently engaged with the task.
Non-participation	9 participants timed out of the study by taking more than 71 minutes, and 14 participants voluntarily left the study part way through. The 149 participants used for our analyses all completed the entire study.
Randomization	Our study involved no allocation into groups, and data from all subjects were analyzed together.

Reporting for specific materials, systems and methods

We require information from authors about some types of materials, experimental systems and methods used in many studies. Here, indicate whether each material, system or method listed is relevant to your study. If you are not sure if a list item applies to your research, read the appropriate section before selecting a response.

Materials & experimental systems

n/a	Involved in the study
☒	☐ Antibodies
☒	☐ Eukaryotic cell lines
☒	☐ Palaeontology and archaeology
☒	☐ Animals and other organisms
☒	☐ Clinical data
☒	☐ Dual use research of concern

Methods

n/a	Involved in the study
☒	☐ ChIP-seq
☒	☐ Flow cytometry
☒	☐ MRI-based neuroimaging
